# Mapping the genetic landscape establishing a tumor immune microenvironment favorable for anti-PD-1 response

**DOI:** 10.1016/j.celrep.2025.115698

**Published:** 2025-05-08

**Authors:** Daniel A. Skelly, John P. Graham, Mingshan Cheng, Mayuko Furuta, Andrew Walter, Thomas A. Stoklasek, Hongyuan Yang, Timothy M. Stearns, Olivier Poirion, Ji-Gang Zhang, Jessica D.S. Grassmann, Diane Luo, William F. Flynn, Elise T. Courtois, Chih-Hao Chang, David V. Serreze, Francesca Menghi, Laura G. Reinholdt, Edison T. Liu

**Affiliations:** 1 The Jackson Laboratory for Mammalian Genetics, Bar Harbor, ME 04609, USA; 2 The Jackson Laboratory, Sacramento, CA 95838, USA; 3 The Jackson Laboratory for Genomic Medicine, Farmington, CT 06032, USA; 4 Single Cell Biology Lab, The Jackson Laboratory for Genomic Medicine, Farmington, CT 06032, USA; 5 OB/Gyn Department, UConn Health, Farmington, CT 06032, USA; 6 These authors contributed equally; 7 Lead contact

## Abstract

Identifying host genetic factors modulating immune checkpoint inhibitor (ICI) efficacy is experimentally challenging. Our approach, utilizing the Collaborative Cross mouse genetic resource, fixes the tumor genomic configuration while varying host genetics. We find that response to anti-PD-1 (aPD1) immunotherapy is significantly heritable in four distinct murine tumor models (*H*^2^: 0.18–0.40). For the MC38 colorectal carcinoma system, we map four significant ICI response quantitative trait loci (QTLs) with significant epistatic interactions. The differentially expressed genes within these QTLs that define responder genetics are highly enriched for processes involving antigen processing and presentation, allograft rejection, and graft vs. host disease (all *p* < 1 × 10^−10^). Functional blockade of two top candidate immune targets, GM-CSF and IL-2RB, completely abrogates the MC38 transcriptional response to aPD1 therapy. Thus, our *in vivo* experimental platform is a powerful approach for discovery of host genetic factors that establish the tumor immune microenvironment propitious for ICI response.

## In brief

Skelly et al. use an *in vivo* murine system to identify host genes whose expression predicts for response to the immune checkpoint inhibitor, anti-PD1. Genetic mapping shows specific epistatic interactions. Blockade of each of the products of two genes completely reverses anti-PD1 response.

## Graphical Abstract

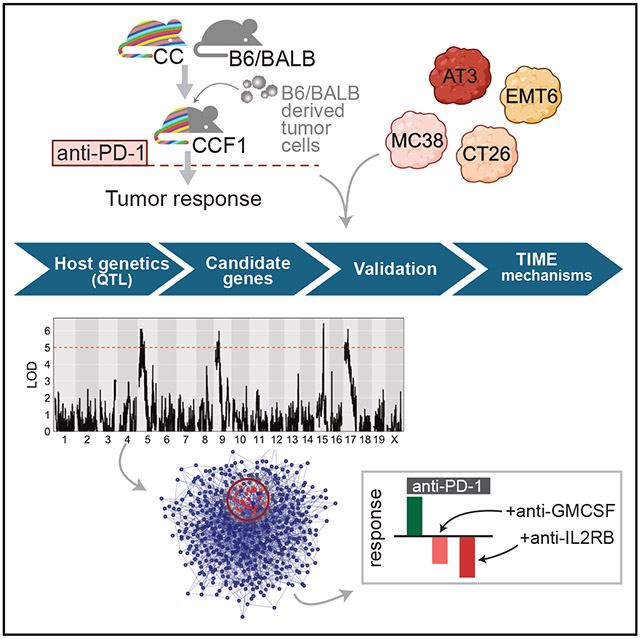

## INTRODUCTION

Abrogating immune checkpoint control has proven to be a powerful therapeutic strategy against cancer, with documented long-term remissions treating refractory metastatic cancers. However, only a subset of patients (10%–40%) have meaningful responses.^1,2^ Cancer cell-intrinsic features such as somatic genetic alterations activating specific oncogenic pathways,^3,4^ the mutational load,^5,6^ the presence and degree of microsatellite instability,^7^ and aneuploidy^8^ can differentially influence the responsiveness of the immune system to cancer.^9-11^ The composition of the tumor-immune microenvironment (TIME), influenced by both intrinsic^12,13^ and extratumoral (e.g., microbiome^14,15^) factors, can in turn affect immune checkpoint inhibitor (ICI) responsiveness.^11,16,17^ Since germline genetic variation has a significant impact on virtually every clinically relevant immunologic trait, including autoimmunity, transplant rejection, response to microbial challenges, and cancer susceptibility,^18-22^ it is only rational to assume that genetic variation in host genes participating in immune responses would alter the TIME, and ultimately modulate anti-tumor responses to ICI treatment.

However, there are few detailed studies of host genetic effects on ICI response. While prior work supports the notion that certain germline genetic variants associated with individual immune genes, including specific HLA subtypes, can alter immunotherapeutic responses,^20,23-31^ these studies are fundamentally limited by the complexity of the clinical patient population under study, and the clear genomic differences of the varied tumors under treatment: every patient’s immune system is different because of host genetic variations; each tumor has a unique genomic profile shaped by its own mutational origins and evolutionary path; each patient’s immune status has been further modulated by environmental factors, the microbiome, age, and prior anti-cancer treatment. Moreover, the logistics of tissue acquisition in clinical trials, and the range of technologies applied (e.g., candidate gene analysis vs. whole-genome/transcriptome approaches) pose statistical and analytical challenges. Taken together, these complexities preclude precise identification of ICI response-modulating genes using standard GWAS approaches in clinical situations.

To address these challenges, we have leveraged the inbred yet highly genetically variable collaborative cross (CC) mouse genetic resource and an F1 breeding strategy to implant syngeneic tumors that have a fixed genomic configuration into mice with variable—yet definable and reproducible—host genetic backgrounds (different CC strains). With this approach, we quantify a significant impact of host genetics in modulating ICI responses across several distinct tumor models, and map distinct quantitative trait loci (QTLs) that both additively and epistatically influence response to anti-PD-1 (aPD1) immunotherapy. Confirming the impact of our observations, we show the abrogation of transcriptional response to aPD1 when several key quantitative trait gene (QTG) products were blocked. Together, these results demonstrate the power of our system for unbiased discovery of genes influencing ICI response.

## RESULTS

### A platform for investigating the genetics of ICI response

**Figure 1.**
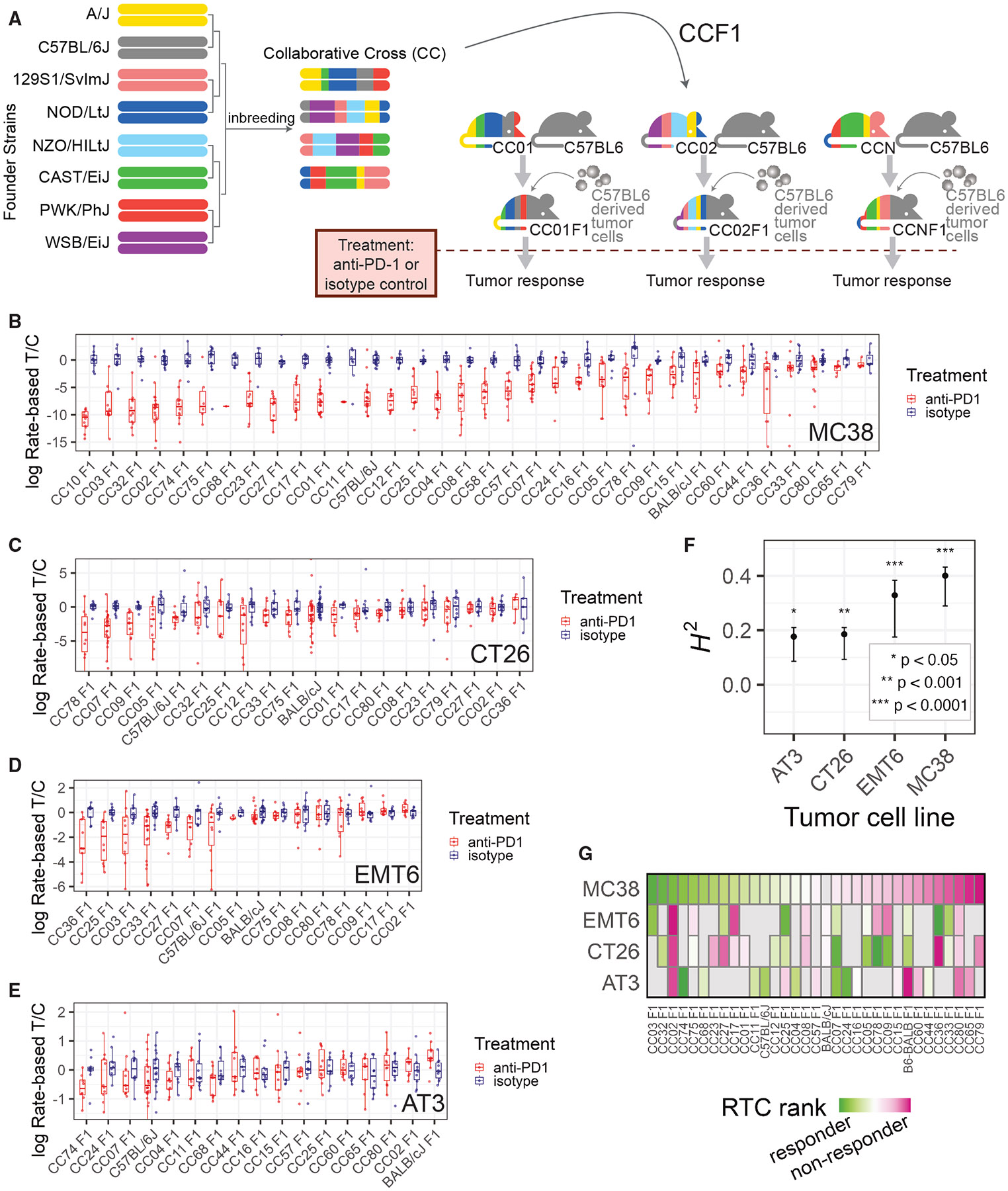
A system for quantifying ICI response across variable host genetics and its application to four implantable tumor models (A) Schematic overview of our mouse CC-based system for measuring ICI response in diverse but replicable genetic backgrounds using a tumor with fixed genetic background. (B–E) Boxplots showing our measure of ICI response, the per-mouse rate-based tumor/control (RTC) computed across strains implanted with each tumor cell line model. Boxes show first quartile, median, and third quartile, while whiskers extend to a maximum of 1.5 times the interquartile range. Blue points and boxes display RTC for isotype control mice, which are approximately centered at zero for all strains, while red points/boxes display values for aPD1-treated mice. A large difference between red and blue boxes for a particular strain would indicate a strong response to ICI. (F) Significant broad-sense heritability $\left(H^{2}\right)$ of ICI response was seen for each implantable tumor cell line model, computed using data from all CCF1 lines profiled with that model. Error bars show 95% confidence interval based on 10,000 bootstrap samples. Asterisks indicate significance of a test for $H^{2}$ greater than chance as indicated in the legend at the bottom right. (G) Heatmap of median log(RTC) values standardized across each tumor model (values are ranked, which is strongly correlated to normalized *Z* score [ρ = 0.96, *p* < 2.2 × 10^−16^]) and plotted across all strains profiled in at least two tumor models. Heatmap demonstrates the differential impact of the CCF1 crosses on aPD1 response depending on the tumor model used. See also Figures S1 and S2.

We leveraged common tumor engraftment models and the CC mouse genetic reference population^32^ to establish an experimental system for identification of host genetic factors influencing ICI response. The major challenge for tumor transplantation models in genetically diverse backgrounds is allogeneic tumor rejection. To overcome this impediment, we used an F1 hybrid approach whereby genetically diverse inbred CC strains were crossed to the inbred genetic background of the transplanted tumor cell line (Figure 1A).^33^ We chose multiple murine tumor cell lines from two genetic backgrounds, C57BL/6J (B6) and BALB/cJ (BALB), covering both mammary gland and colon cancers: MC38 (colon; B6), CT26 (colon; BALB), AT3 (mammary gland; B6), and EMT6 (mammary gland; BALB). These cell lines have been shown to be variably responsive to ICI treatment^34-36^ and provided a balanced mix between different tissues of origin (mammary and colon) and genetic backgrounds (BALB and B6).

First, we constructed host recipients for the MC38 colon cancer line. These recipients were F1 offspring of crosses between B6 and each of 32 inbred CC lines (F1 offspring referred to here as “CCF1,” Figure 1A). The CC is a multiparent, recombinant inbred strain panel derived from 8 inbred founder strains segregating ~40 million genetic variants.^32,37,38^ Each CCF1 tumor recipient mouse carried maternal ancestry from a CC strain together with paternal ancestry from B6. The advantage of the CC resource is that each line carries a fixed set of genetic variants present on haplotype blocks segregating at a frequency of approximately 1/8 (eight founder strains with evenly distributed genetic contributions). This approach produced a set of CCF1 strains with high genetic diversity yet having the experimental replicability of inbred strains. We created a panel of CCF1 recipients for the AT3 cell line in a similar manner, as well as crossing CC strains to BALB to create CCF1 recipients for the CT26 and EMT6 (i.e., BALB derived) lines (outlined in Figure S2).

### Variation in ICI response is heritable in genetically diverse mice

To measure ICI response in individual strains of mice, we developed a method to quantify the magnitude with which ICI treatment retards tumor growth in comparison to treatment with isotype control antibody (per-mouse rate-based tumor/control^39^ [RTC]; see STAR Methods). The RTC metric assumes that tumors grow exponentially and utilizes linear regression modeling of the logarithm of tumor size to derive an estimate of tumor growth rate that is robust to statistical noise and missing data. We found that the variation in RTC within a CCF1 strain is less than that observed between strains (e.g., Figures 1B-1E).

When applied to the MC38 system, a broad range of aPD1 responses from individual CCF1 strains was observed, from nearly all complete responders (CC02 F1) to strains with no discernable response (CC11 F1) (Figure 1B). Across 34 lines (including 32 genetically diverse CCF1 lines, inbred B6, and B6/BALB F1 mice), quantitative genetic analysis revealed that ~40% of the total variation in ICI response in the MC38 model is attributable to genetic background (broad-sense heritability *H*^2^ = 0.40; *p* < 1 × 10^−4^) (Figure 1F). Next, we scanned >15 CCF1 crosses using each of the other 3 murine cancer models (Figures 1C-1E) and found significant heritability for each (Figure 1F): 0.33 for EMT6 (*p* < 1 × 10^−4^), 0.19 for CT26 (*p* = 2 × 10^−4^), and 0.18 for AT3 (*p* = 0.017). Interestingly, each tumor model had a distinct set of responder and non-responder CCF1 strains (Figure 1G). For example, CC02F1 mice are good responders in the MC38 system but poor responders for the other tumor models, and while CC78F1 mice show a good response in the CT26 model, they are non-responders in the EMT6 and MC38 models. This suggests that tumor intrinsic factors influence how host genetics mediates responsiveness to ICI.

The microbiome has been reported to alter the efficacy of ICI therapeutics.^40^ To assess this possibility, we tested two aPD1 responder CCF1 lines (CC02 and CC75) for response with and without pre-treatment with a broad-spectrum cocktail of antibiotics that substantially reduces the gut microbiome and its effects on other phenotypes.^41^ Importantly, we found no diminution of the ICI response to MC38 tumors (Figures S1A and S1B), strongly suggesting that ICI responses in our MC38 CCF1 system are a direct effect of host genetics and not influenced by the microbiome.

Although previous reports of the sequence characteristics of CT26, MC38, and EMT6 exist,^42,43^ we re-sequenced all four cell lines. When the two cell lines with the highest heritability (MC38 and EMT6) were compared with the two with the lowest H^2^, no distinguishing features emerged. MC38, a B6-derived colon carcinoma, had the highest tumor mutational burden (TMB = 112 mutations per Mb; Figure S1C) and is MSI (microsatellite instability) positive.^44^ EMT6, which had the second highest H^2^, has low TMB (18.5 mutations per Mb) and is a BALB-derived mammary carcinoma line. Surprisingly, given the association of TMB and ICI response, the CT26 cell line had an *H*^2^ of only 0.19 (Figure 1C) despite having the second highest TMB (92 mutations/Mb; Figure S1C). Moreover, no characteristic single-base substitution signature or other structural variations distinguished high vs. lower heritability (Figure S1D). Thus, genomic analyses of these four tumor models showed no obvious genomic signature linked to aPD1 response.

### Identification of aPD1 response QTLs and their epistatic interactions

**Figure 2.**
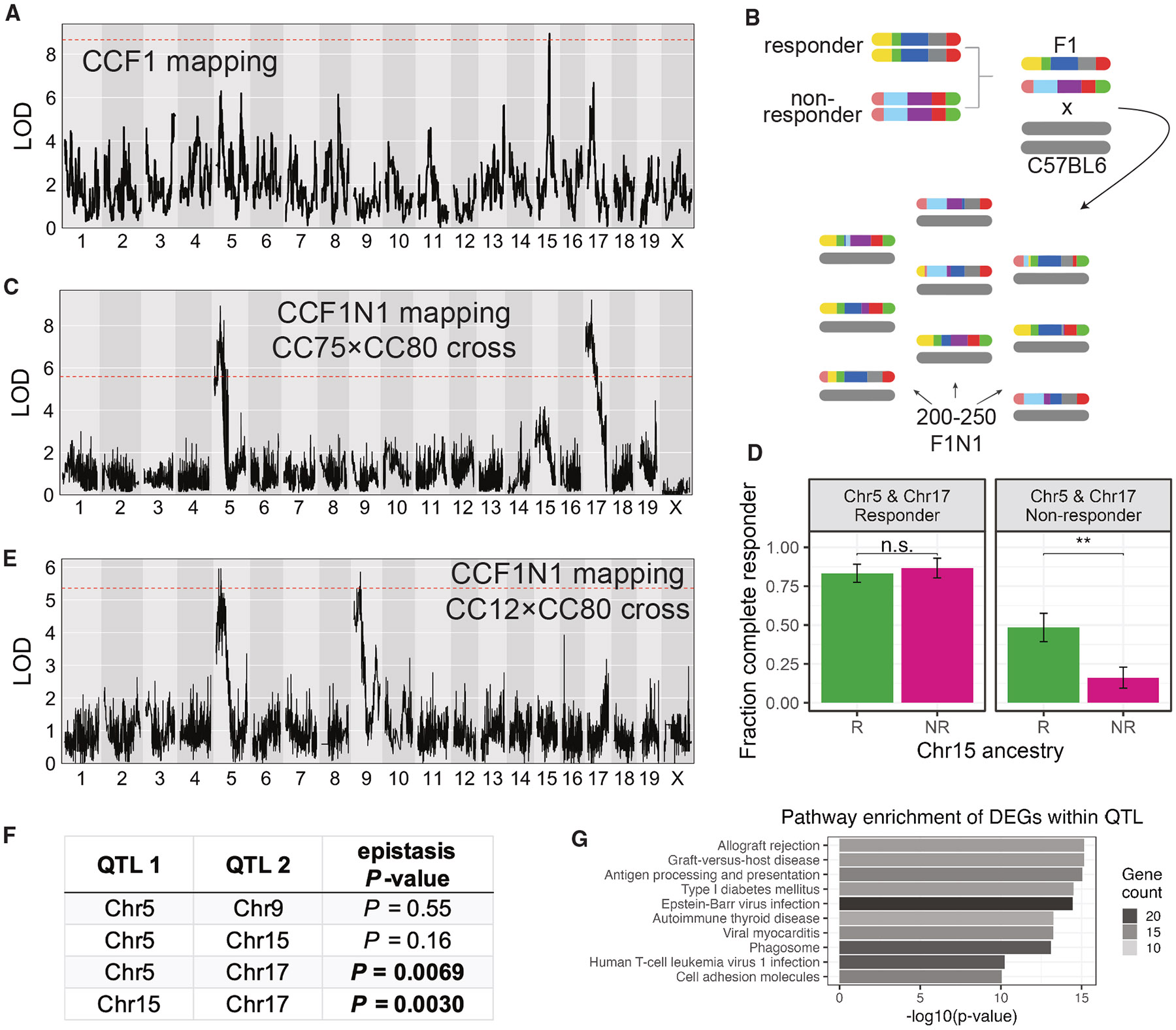
Discovery of ICI response QTLs and their epistatic interactions (A) LOD curve for a genome scan of ICI response in CCF1 lines using the MC38 tumor model. (B) Schematic outlining breeding design to generate a mapping population for CCF1N1 studies. (C) LOD curve for a genome scan of ICI response in CCF1N1 (CC75×CC80) mice using the MC38 tumor model. (D) Illustration of epistatic interactions between QTL in our CCF1N1 (CC75×CC80) mapping population. Three QTLs on mChr 5, 15, and 17 were polarized as responder (R) or non-responder (NR) using haplotype effect estimates from our CCF1N1 mapping. The results show that the responder haplotype on Chr15 exhibited an *in vivo* response mainly when both the Chr5 and Chr 17 haplotypes are in the non-responder configuration. Error bars show mean ± 1 standard error within each group. Wilcoxon test, **p < 0.01; n.s., not significant, respectively. (E) LOD curve for a genome scan of ICI response in CCF1N1 (CC12×CC80) mice using the MC38 tumor model. Since the two CC lines are matched for the mChr 17 haplotype, the mChr 17 QTL is absent. (F) Table reporting results of statistical testing of epistatic interactions between QTLs (see STAR Methods). Pairs of QTLs with significant evidence of interaction are shown with *p* value in bold. (G) Top 10 most enriched pathways among responder vs. non-responder strain differentially expressed genes that are physically located within one of our four QTL intervals. Bar length is proportional to the −log10(*p* value) for enrichment, while bar shading is proportional to the number of genes in each annotated pathway contained within our gene set (DEGs within QTLs). See also Table S1.

We carried out QTL mapping with the MC38 CCF1 model, which had the highest estimated heritability for ICI response (Figure 1F) and the largest dynamic range of responses (Figure 1B). Using 32 CCF1 strains, we identified a locus on mouse Chr15 having significant association with aPD1 response (LOD = 8.9) along with multiple subthreshold peaks (Figure 2A). Since the CCF1 approach has limited mapping power due to the structure of this population and because only 32 CCF1 lines were used,^45^ we sought to refine these subthreshold peaks using a more powerful mapping design. Therefore, we constructed intercrosses between an ICI responder (CC75) and a non-responder CC line (CC80) and mated the offspring of this CC intercross to B6 female mice (a design which we have termed CCF1N1 mapping, schematized in Figure 2B; see also Figure S2). While paternally derived CC strain chromosomes are genetically variable, CCF1N1 mice carry maternally derived chromosomes from the B6 background, which initiates host immunological compatibility with the MC38 model. Moreover, because paternal chromosomes are derived from a cross between responder and non-responder CC lines, this design guarantees that ICI response-modulating genetic variation segregates in the mapping population.

In the CCF1N1 design, each mouse is genetically unique thus cannot be normalized to an isotype control group. For QTL mapping in this population (*n* = 249 mice), we mapped either un-normalized RTC or a discrete response classification (STAR Methods). We identified significant QTLs for aPD1 response on mChr5 and mChr17, which originally appeared in CCF1 mapping as statistical subthreshold peaks (Figures 2A vs. 2C). While the original CCF1 mapping showed a prominent mChr15 QTL, this peak, although present, lost statistical significance in the subsequent CCF1N1 crosses with the emergence of QTLs on mChr5 and 17. We hypothesized that intercross-specific combinations of haplotypes at these QTLs could significantly modulate the impact of a given locus. Indeed, we observed epistatic interactions between QTLs: the QTL on mChr15 did not affect response when mice carried responder haplotypes at mChr5 and 17 QTL, yet the mChr15 QTL had a strong effect on response in mice carrying non-responder haplotypes at these loci (Figure 2D).

This result suggested that additional QTLs might be uncovered in specific genetic contexts while being masked by epistatic effects in other contexts. We tested this hypothesis by initiating a CCF1N1 study selecting lines for the CC intercross that had matched ancestry at the mChr17 QTL, which encompasses the MHC locus including MHC class I and II molecules. We asked whether we could identify additional MC38 response-modifying QTLs by generating CCF1N1 mice (*n* = 237) using a responder (CC12) strain and non-responder strain (CC80) with matched ancestry at the mChr17 QTL but segregating variable haplotypes (responder vs. non-responder) at the mChr5 and 15 QTLs. With this approach, as expected, the mChr17 QTL was eliminated while the QTL on mChr5 was detected. However, consistent with our speculation about potential masking of QTL by epistasis, we observed a new and significant QTL on mChr9 (Figure 2E).

Because of the structure of the genetic crosses, where multiple QTLs segregated in a cross, we could statistically quantify the extent of epistasis between loci using logistic regression modeling (Figure 2F). For the first CC80×CC75 CCF1N1 experiment, we did not observe evidence for 3-QTL interaction (*p* = 0.34); however when pairwise interactions between QTLs were assessed, interactions between mChr15-17 and mChr5-17 QTL were statistically significant while the Chr5-15 QTL interactions were not (Figure 2F). An independent examination of the second CC12×CC80 CCF1N1 experiment suggested no epistasis between the Chr5 and Chr9 QTL (Figure 2F).

### Immune genes within QTL are the key drivers of ICI response in the CCF1 MC38 system

Since the four QTLs span large segments of the genome (approximately 5–50 Mb each), the total number of candidate QTGs is high (*n* = 2,793; Table S1). We obtained bulk transcriptional profiles of MC38 tumors (*n* = 23) from three responder strains (CC01 F1, CC02 F1, CC75 F1) and three non-responder strains (CC36 F1, CC79 F1, CC80 F1) treated with either isotype or aPD1. Of 22,419 genes with detectable expression in these tumors (≥5 reads across samples), 2,395 (10.7%) were significantly differentially expressed genes (DEGs) (Table S1) between responder and non-responder tumors (FDR = 5%) but only 1,255 of these were in the QTL.

Given the fundamental importance of immunobiology to ICI response, we sought to understand the degree to which genes with known immune-related functions were reflected in the DEGs within QTLs. We leveraged the InnateDB resource^46^ to compile a systematically annotated list of genes (*n* = 8,400) with immune function. Overall, our QTLs harbored a slightly higher fraction of immune-related genes compared with the genomic background regardless of gene expression (Fisher’s test, *p* = 0.05). However, the 160 DEGs within our QTLs distinguishing responders from non-responders (Table S1) were very strongly and significantly enriched for immune function compared with non-DEGs within QTLs (58% vs. 31%; Fisher’s test *p* = 3.5 × 10^−11^).

Top enriched KEGG pathways (all *p* < 1 × 10^−10^) of DEGs within QTLs included antigen processing and presentation, allograft rejection, and graft vs. host disease (Figure 2G), which is consistent with the enhancement of responder strain anti-tumor (anti-self) immune responses induced by ICIs. Other top enriched KEGG pathways (all *p* < 1 × 10^−10^) included pathways related to viral infection (Epstein-Barr virus, myocarditis, human T cell leukemia virus) or autoimmune processes (type 1 diabetes, thyroid disease; Figure 2G; Table S1). These results likely reflect the role of cytotoxic T lymphocyte (CTL) and interferon responses in immune regulation of the TIME^47^ and the balance between the response against self-antigens and the destruction of malignant cells in a successful immune response to cancer.^48^ By contrast, there were no statistically significant enriched pathways among the non-immune DEGs within QTLs suggesting the absence of cohesive functions in these DEGs.

Among DEGs with immune function located within QTLs, over half were derived from the mChr17 QTL (*n* = 51 [mChr17], 26 [mChr9], 12 [mChr15], and 4 [mChr5]). While genes in the mChr17 QTL were strongly enriched for pathways representative of antigen presentation, autoimmunity, and interferon responses, none of the DEGs in each of the remaining QTLs associated strongly with a specific immune function (i.e., no enrichment of specific pathways with *p* < 0.001). The mChr17 QTL appears to be a special case since it encompasses the entire MHC locus, which includes MHC class I and II as well as immune genes such as TNF and lymphotoxin.

Since there are DEGs outside the QTLs that are also immune related, we asked if there were differences between immune DEGs within QTLs and those outside QTLs. While immune DEGs within all QTLs were strongly enriched for pathways linked to cellular immunity and immunotherapy responses (antigen presentation, autoimmunity, interferon responses, Th1/Th2/Th17 cell differentiation, and T cell receptor signaling), immune DEGs outside the QTL were enriched for pathways that were not as obviously associated with anti-cancer immunity (e.g., response to bacterial infection, B cell receptor signaling, apoptosis). Together, these results suggest that there is functional coherence in the major host genetic drivers within our QTLs for ICI response and that these drivers interact with each other to establish a responsive TIME.

### Immunophenotyping reveals specific characteristics associated with the ICI-responsive tumor immune microenvironment

**Figure 3.**
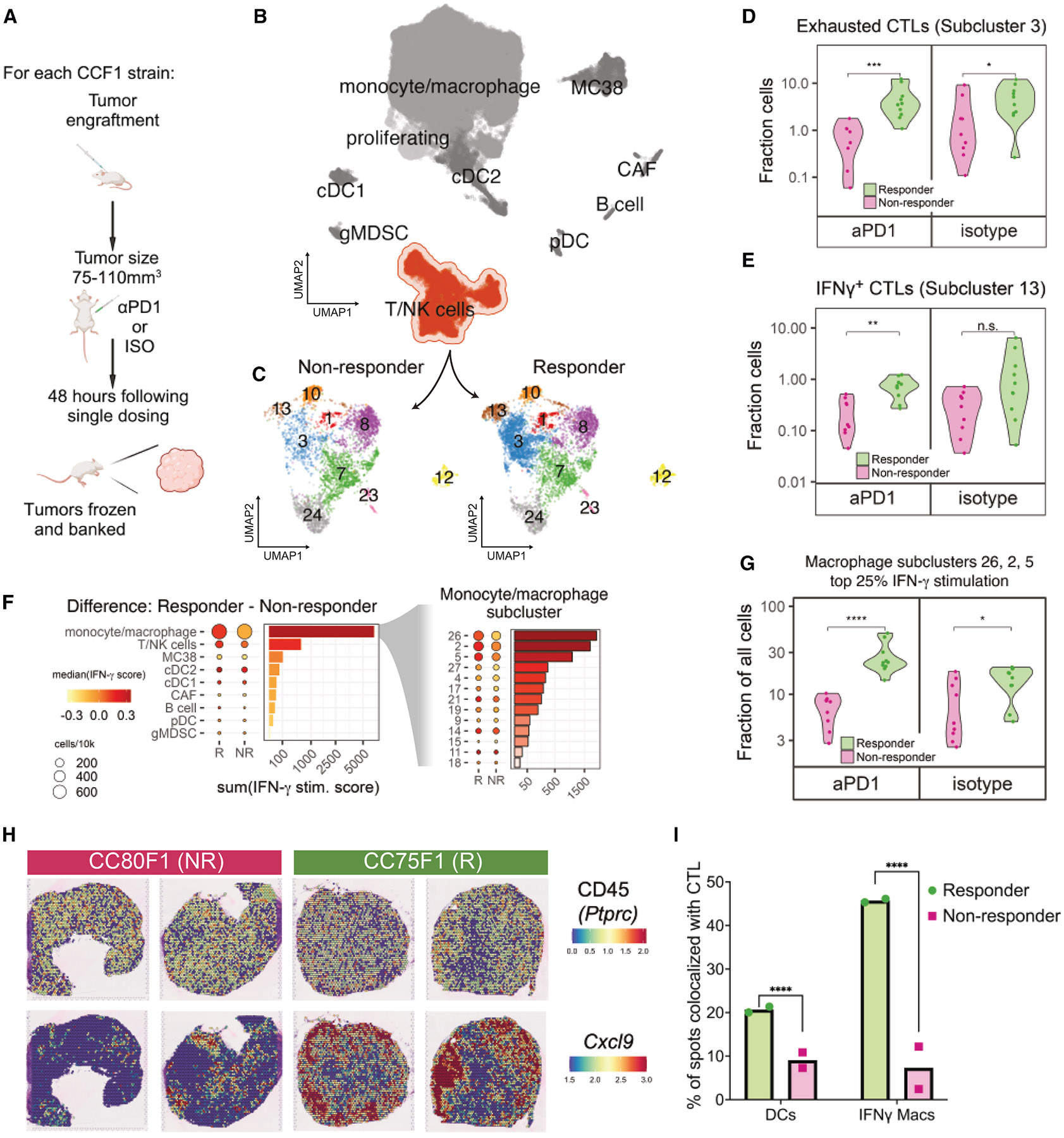
Immunophenotyping reveals enrichment and co-localization of CTLs and IFN-γ-stimulated macrophages in responder strain MC38 tumors (A) Overview of single dose paradigm where tumors are harvested 48 h after a single dose of aPD1 or isotype control in order to capture the initial effects of treatment. (B) UMAP plot depicting an overview of heterogeneity in single-cell transcriptomes derived from responder and non-responder strain tumors and highlighting the T/NK cell cluster. (C) Subclustering of the T/NK cell cluster reveals transcriptionally distinct cell subsets showing enrichment of subclusters 3 and 13 in responding tumors. (D) Fraction of exhausted CTLs (cluster 3) as portion of all cells in responder and non-responder strain groups, Wilcoxon test, ***p < 0.001, *p < 0.05, respectively. (E) Fraction of IFN-γ^+^ CTLs (cluster 13) as portion of all cells in responder and non-responder strain groups, Wilcoxon test, **p < 0.01; n.s., not significant, respectively. (F) Expression of an 18-gene signature reflecting IFN-γ^+^ stimulation applied to all cells in each cluster (left) or macrophage subcluster (right), with dot size proportional to the relative number of cells of each type, dot color relative to the median IFN-γ^+^ stimulation score, and bars depicting the difference in summed pathway score between responder and non-responder cells within each (sub)cluster. (G) Fraction of IFN-γ-stimulated macrophage clusters (clusters 2, 5, and 26) as a portion of all cells in responder and non-responder strain groups, Wilcoxon test, ****p < 0.0001, *p < 0.05, respectively. (H) Visium spatial transcriptomics images showing expression of CD45 (*Ptprc*) (top row) and *Cxcl9* (bottom row) in spatially resolved spots of tumors harvested from responder (CC75F1) and non-responder (CC80F1) mouse tumors. Hematoxylin and eosin stains showed no evidence of necrosis in these tumor sections. (I) Percentage of *Ptprc*^+^ spatial transcriptomics spots co-expressing either macrophage lineage markers (*Adgre1, Cd68, Itgam*, and *Cxcl9*) or dendritic cell markers (Batf3 and/or Zbtb46) along with CTL markers (*Thy1, Cd8a*, and *Gzmb*) across all tissue regions in responder vs. non-responder strain tumors. ****p < 0.0001, permutation test (see STAR Methods). See also Table S2.

We next sought to define the differences in the TIME between responder vs. non-responder CCF1 strain MC38 tumors. To accomplish this, we first examined changes in cellular composition within tumors. To this end, we selected three consistent responder strains (CC01 F1, CC02 F1, and CC75 F1) and three non-responder (CC36 F1, CC79 F1, and CC80 F1) strains for immunophenotyping based on their response in our initial screen (Figure 1B). Forty-eight hours after a single dose of either aPD1 or isotype control, when the tumors show no macroscopic changes in size between treatment groups, we harvested MC38 tumors and characterized their cell composition using both single-cell RNA sequencing (scRNA-seq) and flow cytometry (Figure 3A). This approach ensured that any changes we quantified were not due to volume changes in the tumors.

Flow cytometry revealed no significant difference in the overall infiltration of CD45^+^ immune cells between tumors from responder (*n* = 28) and non-responder (*n* = 25) strain mice either in the isotype-treated or in the single dose aPD1-treated groups (Table S2). Specifically, the immune cellular composition of the MC38 TIME was constant with CD11b^+^F4/80^+^ macrophages dominating at ~70% of CD45^+^ cells, followed by F4/80^−^CD3e^+^ T cells (~20% of CD45^+^ cells), and then dendritic cells (DCs) (~10% of CD45^+^ cells) regardless of the treatment or the response group (Table S2). These proportions are similar to those previously described for the MC38 model.^49^ However, our data showed a significant increase in intratumor MC38 antigen-specific MHC class I restricted tetramer-specific CTL and PDL1^high^MHC class II^high^ macrophages in MC38 tumors of responder CCF1 strains (Table S2).

To further dissect cell heterogeneity in these tumors, we built an immune-focused cellular atlas of MC38 tumors using scRNA-seq. We used MC38 tumors from our single ICI dose protocol (Figure 3A), capturing a range of responder (*n* = 20; tumors from CC01 F1, CC02 F1, and CC75 F1) and non-responder (*n* = 18; tumors from CC36 F1, CC79 F1, and CC80 F1) strain tumors. We profiled tumors using the 10X Genomics Chromium platform after sorting to enrich for the CD45^+^ compartment (STAR Methods) recovering approximately 76,000 cells from responder and non-responder CCF1 strain tumors.

Using standard methods to cluster single-cell transcriptional profiles, we identified nine major cell populations (Figure 3B): in addition to MC38 cells (*Shisal2b*^50^), monocyte/macrophages (expressing *Adgre1, Apoe, Lyz2*) were the predominant tumor-associated immune component, followed by T/NK cells (*Cd3e, Nkg7, Ifng*), with B cells (*Cd79a, Igkc, Ms4a1*), conventional DCs (cDC1 *Xcr1, Clec9a, Irf8*; cDC2 *Cd209a, Cd80, Flt3*), plasmacytoid DCs (pDC *Ccr9, Klk1, Siglech*), cancer-associated fibroblasts (*Col1a1, Bgn, Postn*), and granulocytic myeloid-derived suppressor cells (*Gsr, Retnlg, S100a9*) accounting for a significantly smaller proportion of the tumor-associated stroma. These were present in approximately the same proportion as was found in the flow cytometry data (Table S2).

Subclustering of T/NK cells (Figure 3C) revealed two CTL subclusters that were significantly enriched in responder strain MC38 tumors (Table S2; Figures 3D and 3E). Gene expression analysis of these CTL subgroups (scRNA-seq subclusters 3 and 13) revealed a cassette of marker genes that identified CTLs (*Cd3e, Cd8a, Cd8b1*), and either markers of exhaustion (subcluster 3; *Tox, Lag3*, and *Pdcd1* [PD-1]),^51^ or cytotoxicity (subcluster 13; *Ifng, Gzmb, Prf*, and *Tbx21*). The cytotoxicity subcluster (13) exhibited the highest expression of *Ifng* (IFN-γ) when compared with other cells in the dataset (>34-fold).^52^ Proportions of these CTL clusters were higher in responder tumors regardless of treatment (Figures 3D and 3E). Thus, enrichment of *Tox*^+^ exhausted-like and *Ifng*-expressing CTLs defining the responder strain TIME were established prior to ICI exposure rather than in response to aPD1 therapy.

Because *Ifng*^+^ CTLs were enriched in responder strain tumors, we examined which cells might be stimulated by IFN-γ in responder strain tumors. To assess stimulation by IFN-γ, we used a previously published^53^ 18 gene IFN-γ-related mRNA profile (IFN-γ response signature, “Merck18”). We compared cells using this signature and found that macrophages showed a prominent difference between responder and non-responder samples (Figure 3F, left). Three subclusters—2, 5, and 26—contained the bulk (82%) of macrophages with high IFN-γ response signature scores (Figure 3F, right), suggesting that these macrophage subpopulations were primarily responding to IFN-γ stimulation. Cells within macrophage subclusters 2, 5, and 26 that had high IFN-γ response signature scores (upper quartile) were significantly enriched in responder vs. non-responder strain tumors (Table S2 ; Figure 3G). PD-L1 and MHC class II are IFN-γ-inducible genes,^53^ and our FACS data using these markers confirmed that a PD-L1^high^MHC class II^high^ M1-like macrophage population^54^ was enriched in the responder strain TIME (Table S2).

These data suggested a potential interaction between CTLs and macrophages. Using 10X Genomics Visium spatial transcriptomics, we asked if the IFN-γ-stimulated (*Cxcl9*^+^) macrophage population and CTLs were distributed differently between tumors from responder (CC075F1, *n* = 2) vs. nonresponder strains (CC080F1, *n* = 2) 2 days after treatment with a single dose of isotype antibodies to represent a pre-aPD1-treated tumor TIME state. Spatial expression of *Ptprc* (CD45), the pan-immune cell marker, suggested that both responder and non-responder tumors were similarly diffusely infiltrated by immune cells (Figure 3H). In contrast, examining the IFN-γ-responsive marker *Cxcl9*^53^, responder tumors displayed substantially more positive spots, suggesting intratumor clusters of *Cxcl9*^+^ monocyte/macrophages (Figure 3H). An examination of the CTL marker, *Cd8a*, similarly showed an increase in responder tumors. By computing the fraction of *Ptprc*^+^ spots co-expressing either macrophage markers (*Adgre1, Itgam, Cd68*, and *Cxcl9*) or DC markers (*Batf3* and/or *Zbtb46*) and expressing the CTL markers *Thy1* and *Cd8a*, we found that responder strain tumors showed prominent colocalization of *Cxcl9*^+^ macrophages with CTLs: responder strain tumors have >4× CTL-macrophage co-localized spots and 2× more DC-CTL interactions than non-responder strain tumors in this pre-treated state (Figure 3I).

### Linking mouse ICI genes with human genetic and transcriptional signals

After establishing the importance of immune genes in our QTLs, and the differences in TIME between responders and non-re-sponders, we sought to determine whether our findings align with the human condition. In humans, HLA heterozygosity positively correlates with survival in patients treated with ICIs.^25^ The importance of the orthologous locus (MHC) in mouse is underscored by the robust detection of a QTL at this locus, the engagement of this mChr17 QTL in epistatic interactions with mChr 5 and 15 QTLs, and that 9 of the top 100 response-associated DEGs within QTL were MHC genes. The human homologs of these top genes were both HLA class I (HLA-A, HLA-E, HLA-F) and class II genes (HLA-DMA, HLA-DMB, HLA-DQA1, HLA-DQB1, HLA-DRA, HLA-DRB5). Mice that are homozygous for shared B6 and 129 MHC haplotypes (H2^b^ including H2-K^b^) exhibit the best responses as 129 is haploidentical (including H2-K^b^) with B6 at the MHC locus. Since p15E-H2^Kb^-restricted CTL responses are immunodominant and induced by MC38^55^ (STAR Methods), homozygosity of H2^Kb^ likely increases the overall presentation of the immunodominant MHC-I-restricted p15E peptide, which would increase activation and proliferation of H2Kb-restricted CTLs. Indeed, H2-K^b^-restricted tetramer analysis on responder vs. non-responder tumors after a single dose of aPD1 revealed that responders had a 10-fold greater frequency of H2-K^b^-restricted MC38-specific (against the p15E antigen) CTL response than non-responder tumors (Figure S3A). Thus, the strong response in H2^Kb^ homozygous (B6/129) mice is likely due to more efficient presentation of the MC38 specific, H2-K^b^-restricted antigen and is unique to the MC38 experimental system, a situation that is not likely to occur in most human cancer conditions. Using CCF1 mice carrying other MHC haplotypes (excluding the NOD strain since it is known to have deficits in the maturation and function of antigen-presenting cells^56^), we computed a heterozygosity score for the MHC locus using 84,000 single-nucleotide variants genotyped in the eight inbred CC founder strains. Mirroring the human data, heterozygosity at the MHC locus was associated with improved ICI response as measured by our RTC metric (Figures S3B and S3C).

**Figure 4.**
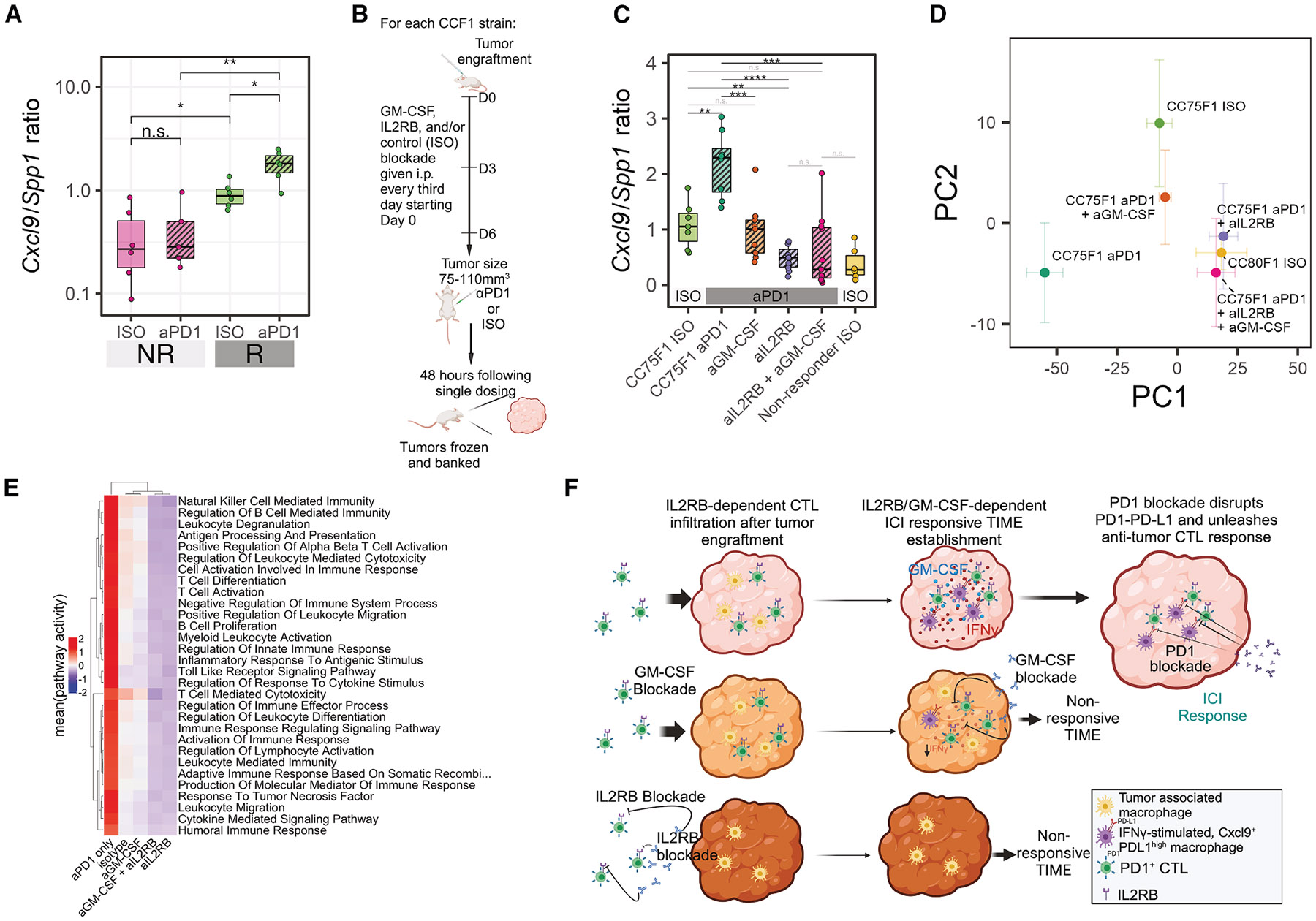
*In vivo* blockade of GM-CSF and IL2RB reverses the transcriptional signature of aPD1 response (A) *Cxcl9/Spp1* ratio in MC38 bulk RNA-seq samples from responder and non-responder strain tumors. Boxes show first quartile, median, and third quartile, while whiskers extend to a maximum of 1.5 times the interquartile range. Wilcoxon test, **p < 0.01, *p < 0.05; n.s., not significant. (B) Schematic overview of the blocking antibody dosing protocol and strategy for assessing tumors before macroscopic changes in size. (C) *Cxcl9/Spp1* ratio computed using RNA-seq gene expression profiling of CC75 F1 MC38 tumors collected as in (B). Boxes show first quartile, median, and third quartile, while whiskers extend to a maximum of 1.5 times the interquartile range. Wilcoxon test, ****p < 0.0001, ***p < 0.001, **p < 0.01, *p < 0.05; n.s., not significant. (D) Principal-component (PC) plot showing the positioning of each group of samples within the first two PCs. CC75 ISO is the tumor in a responding genetic cross treated with isotype control antibodies, whereas CC80 ISO is the tumor in a non-responding genetic cross treated similarly. Principal-component analysis was performed using batch-corrected RNA-seq profiles (STAR Methods) with error bars showing +/− 1 standard error within each group. (E) Heatmap of enriched immune-related pathways among the experimental groups profiled in this blocking antibody experiment showing reversal of immune signatures. Pathway activity was computed based on leading edge genes identified by gene set enrichment analysis (STAR Methods) and constituted the mean of standardized (per-gene, across all samples) gene expression. (F) Model describing the effects of IL2RB and GM-CSF blockade on the MC38 TIME and response to aPD1. See also Figures S3 and S4.

A recent study of human patients identified macrophage polarity defined by the expression ratio of the genes *CXCL9* and *SPP1* (but not by conventional M1 and M2 markers) as having a strong prognostic association with survival in human cancers, as well as associating with response to aPD1.^57^ To test whether our MC38 system recapitulated this association found in humans, we examined bulk transcriptomes of 23 MC38 tumors from responder and non-responder strain CCF1 mice treated with aPD1 or isotype control using the single-dose protocol previously described. *Cxcl9* and *Spp1* expression in these tumors revealed that the MC38 tumors treated only with isotype control antibody in responder CCF1 strains showed a significantly greater *Cxcl9/Spp1* ratio than those from non-responder strain mice (Figure 4A). Intriguingly, a single dose of aPD1 treatment further augmented the *Cxcl9/Spp1* ratio in responder strain tumors by >2-fold, whereas there was no change in this ratio in non-responder strain tumors. This resulted in a much greater difference in the *Cxcl9/Spp1* ratio in responder strain MC38 tumors after treatment than non-responder strain tumors (Figure 4A). These results demonstrate that the MC38 CCF1 system is not only a cross-species validation of the predictive value of the reported *Cxcl9/Spp1* ratio for ICI anti-tumor response, but also suggests that a TIME propitious for ICI response in our CCF1 system is reflective of the human condition.

### *In vivo* blockade of GM-CSF and IL2RB reverses the transcriptional signature of aPD1 response

Using our CCF1/CCF1N1 approach we have uncovered highly plausible candidate drivers that shape an ICI-favorable TIME in both mice and humans. These top response-associated genes were not expressed focally in any one immune cell type, and did not group within a single pathway, suggesting that broad cellular and genetic networks are responsible. This raised the question of whether simultaneous perturbation of multiple QTGs is required before the aPD1 response can be altered. To address this question, we examined two candidate QTGs on mChr15, *Csf2rb* (GM-CSF receptor) and *Il2rb* (IL-2 receptor β subunit). These genes ranked within the top 50 DEGs located inside QTLs, and had available validated murine reagents. The addition of their cognate cytokines, GM-CSF and IL-2 (i.e., IL2RB-targeted pegylated form), have been noted to enhance ICI response in both preclinical and some clinical studies.^58,59^ In our MC38 single-cell transcriptomics, the genes encoding subunits comprising the high-affinity IL-2 receptor (*Il2ra, Il2rb, Il2rg*) and the IL-15 receptor (*Il15ra, Il2rb, Il2rg*) were co-expressed in a minority of cells but significantly enriched in CTL clusters (binomial test, *p* < 1 × 10^−10^) suggesting that the functional receptor complexes are present in the appropriate immune cell populations in our system. Therefore, our genetic analyses suggest that blockade of the receptors or the cytokines should attenuate aPD1 response in a responder genetic background. A single dose of aPD1 can dramatically augment the *Cxcl9/Spp1* ratio in responder mice (Figure 4A), therefore we used this ratio as a surrogate biomarker of aPD1 response. We first applied a GM-CSF blocking antibody to MC38 tumor-bearing responder mice (CC75×B6) using standard protocols^60^ (Figure 4B) and assessed the *Cxcl9/Spp1* ratio 48 h after administration. While the addition of aPD1 significantly augmented the *Cxcl9/Spp1* ratio in MC38 tumors, the co-administration of aPD1 + anti-GM-CSF (aGM-CSF) drove the ratio back to baseline levels (Figure 4C). When we directly blocked the IL2RB receptor using anti-IL2RB antibody (aIL2RB),^61^ the *Cxcl9/Spp1* ratio was reduced even further to levels similar to those found in tumors of genetic non-responders (Figure 4C) and intratumor CTL infiltration appeared to be compromised (Figure S4, schematic in Figure 4F). When the two blocking antibodies were administered together with aPD1, the ratio declined even further but this difference was not statistically significant.

We explored pathway enrichment of genes differentially expressed in pairwise comparisons between any of the treatment groups. Among the immune-related enriched pathways, all were most highly expressed in the mice administered aPD1 only, with intermediate expression of these pathways in the mice administered isotype control or aGM-CSF + aPD1, and lowest expression in mice where IL2RB receptor was blocked (Figure 4E), following the rank-ordering observed for the *Cxcl9/Spp1* ratio (Figure 4C). Using a whole-transcriptome approach and assessing the changes post treatment of 2,324 responder vs. non-responder DEGs using principal-component analyses, we found that, indeed, the co-administration of aGM-CSF with aPD1 returned the transcriptional configuration in CC75×B6 tumors to the responder baseline (i.e., CC75×B6 treated with isotype control), while aIL2RB treatment transformed the aPD1-induced transcriptome to the configuration of a genetic non-responder (CC80×B6 treated with isotype control; Figures 4D and 4F). These data not only validate our genetic discovery platform but point to an important biological possibility: while our QTL/QTG analysis implicated dozens of genes shaping the ICI response propitious TIME, perturbation of a single gene in this network is capable of completely disrupting the response transcriptional outcome.

## DISCUSSION

Murine systems to study host genetics of ICI response have, until recently, also been lacking due to the absence of genetic diversity.^33^ In a recent study, Hackett et al.^62^ identified a locus on mChr13 that modulated response of the transplantable B16 melanoma cell line to combined aPD1 and anti-CTLA4 (aCTLA4) treatment. They crossed B6 with a panel from the diversity outbred (DO) resource and used 142 F1 crosses as a mapping population, which pointed to the prolactin family as a candidate gene family. Although this elegant study also highlighted a role for host genetics in ICI response, the outbred nature of the DO resource did not allow gene-based functional validation within the appropriate genetic context.

We have resolved this experimental problem by crossing mouse lines with defined genetic variability, the CC strains,^32,37,38^ with the inbred strain specific to the murine tumor models for ICI response. These genetic maneuvers, which form the basis of our CCF1/CCF1N1 platform, overcome allogeneic rejection while introducing sufficient genetic heterogeneity for mapping studies. Importantly, this system significantly reduces experimental variables by restricting the analysis to single tumor cell lines grown to a consistent size *in vivo*, given a single drug (aPD1), with animals in living conditions engineered to minimize the impact of diet and housing as confounding factors. In this manner, host genetic diversity becomes the key variable affecting ICI response. In addition, the ability to fix tumor characteristics allows for the deep investigation of the intratumoral cellular characteristics linked to the specific host genetics. Both are key characteristics of this CCF1 system that cannot be achieved in human studies. Importantly, any biological finding can be replicated and further examined using the same replenishable CCF1 crosses, something that cannot be accomplished with the DO mapping approach (outlined in Figure S2).

We have shown that the broad-sense heritabilities of ICI response were all statistically significant ranging from 18% to 40% across the four tumor cell lines used covering two distinct cancers (colon and breast) derived from two different genetic backgrounds (B6 and BALB). While it is difficult to estimate the clinical impact based on *H*^*2*^ statistics, in the MC38 model with the highest *H*^*2*^ (0.40) we saw the complete range of aPD1 response, from no response to complete response only by adjusting the host genetic background. The levels of heritability we observed for ICI response fall broadly within a range that has been observed for many other human quantitative traits: heritability of coronary heart disease was estimated to be 38% for females and 57% for males,^63^ and 37% for major depressive disorders.^64^ The CCF1 lines that showed the best and the worst aPD1 outcomes largely differed between the different tumor models (see Figure 1G), suggesting that either different response QTLs, different genes within our characterized QTL, or different epistatic QTL interactions define the optimal response for each tumor model.

While pairwise epistasis between markers in human studies can be achieved using standard GWAS approaches, the sample size requirements to detect such interactions are daunting. Using controlled specific intercrosses designed from our original CCF1 screen, we were able to uncover epistatic interactions between QTLs on mChr15 and mChr17, and between mChr17 and mChr5 in a highly efficient manner (number of mice, *n* < 300). That mChr17 appears to be a genetic “organizer” through epistatic interactions with both mChr5 and 15 is not unexpected: mChr17 harbored the greatest number of candidate QTGs of any of the QTLs, and also contains the murine MHC locus, which is responsible for the antigen processing and presentation functionality that is core to many relevant cellular immune functions. Our work further identified the contribution of MHC heterozygosity to ICI response, which is consistent with recent human findings: patients with cancer treated with ICIs showed better survival when heterozygous at HLA class I genes compared with those homozygous at any one of these loci.^25^ This was bolstered by large cohort studies of over 500,000 individuals that showed again HLA heterozygosity was significantly protective for the development of lung cancer.^65^

A major question of any animal model system is how relevant it is to the human condition under study. First, we found that the IFN-γ-stimulated TIME associated with ICI response in the CCF1 crosses mirrored that seen in human tumors responding to ICI treatment.^66^ Similar to our findings demonstrating enrichment and close proximities of CTL and *Cxcl9*^+^*Cd274*^+^ (PD-L1) macrophages in responder strain tumors, patients with NSCLC or metastatic colorectal cancer tumors responding to PD1 pathway blockade have higher numbers and spatial proximities of CD8^+^ and PD-L1^+^ cells as quantified by the Immunoscore-IC score.^67,68^ Moreover, we found that responder strain tumors showed a distinct component shift to *Ifng* or *Pdcd1* (PD1)-expressing CTLs and IFN-γ-activated *Cxcl9*- and PD-L1-expressing macrophage subsets, reflecting core immunological aspects of the response-propitious TIME also previously described in patients.^69^ Finally, we showed that the *Cxcl9/Spp1* ratio that was significantly associated with improved outcome in human cancers was also predictive of response in the MC38 CCF1 system. Our work, however, extends these findings by strongly suggesting that a large portion of the ICI response-promoting TIME is under host genetic control.

In contrast to other murine systems, our CCF1 approach allowed us to specifically test factors that will only alter the TIME with a single, genomically defined syngeneic tumor. The *in vivo* functional blockade of two top candidate QTG pathways, GM-CSF (*Csf2rb*) and IL-2/IL-15 (*Il2rb*) resulting in the complete reversal of the aPD1 transcriptional response validates our mapping system as a relevant discovery platform. Intriguingly, our genomic investigations shed further light as to the nuances of their biology. While aGM-CSF completely reversed the effects of aPD1 back to the untreated MC38 tumor configuration in the CC75×B6 responder genetic background, aIL2RB went further and completely reverted the transcriptional profile of the aPD1-treated tumors back to its baseline in the genetic non-responder (CC80×B6) host. This suggests that there may be a hierarchy of effect where cytokine pathways (such as the IL2R/IL15R system) driving the T cell compartment may play a more foundational role in establishing a response propitious TIME, than GM-CSF, that drives the myeloid compartment. Our identification of *Il2rb*, the β subunit of the multimeric IL2R system, and not the other IL2R components raises an intriguing possibility. Since the IL2RB can also associate with IL15RA and IL2RG in forming a highly functional heterotrimeric IL-15 receptor complex, this suggests that the IL-15 system may also be involved with our identified candidate, which, if corroborated, could explain some of the variances in the clinical trials involving combination pegylated IL-2 and ICIs. Indeed, the genes encoding the three subunits comprising both the high-affinity IL-2 receptor (*Il2ra, Il2rb, Il2rg*) and the IL-15 receptor (*Il15ra, Il2rb, Il2rg*) were co-expressed and significantly enriched in CTL clusters in responding MC38 tumors, suggesting, at least, that the biochemical machinery is present for both signaling pathways in the major effector cells. This CCF1 system provides the platform to dissect these possibilities.

Taken together, our findings identified specific interacting host genes that establish an ICI responsive TIME in mice. In addition, we show that these results are translationally relevant. Unlike other experimental systems, our approach identifies specific genetic backgrounds suitable for testing agents converting non-responders to responders, and vice versa, for any experimentally tractable murine adoptive transfer tumor model. Such a platform will dramatically expand the experimental possibilities for discovering more precise ICIs and dosing protocols.

## STAR★METHODS

### EXPERIMENTAL MODEL AND STUDY PARTICIPANT DETAILS

#### Animal models

All mouse lines used in this study were obtained from sources listed in the key resources table. As detailed below, all mice were female and tumor cell lines were injected at age 8–12 weeks. Female mice were exclusively used to ensure immunological compatibility with the female-derived tumor cell lines, preventing potential rejection mediated by sex-specific antigens that could confound interpretation of tumor-immune interactions. All animal procedures were performed in accordance with protocols approved by the Institutional Animal Care and Use Committee of The Jackson Laboratory (IACUC-16013-1). All experiments were conducted in compliance with the Animal Welfare Act, the Guide for the Care and Use of Laboratory Animals, and other applicable federal and institutional regulations and policies. The study was conducted following the ARRIVE guidelines for reporting animal research.

#### Culturing of cancer cell lines

Among the four murine cancer cell lines, CT26 and EMT6 were obtained from ATCC (https://www.atcc.org), AT3 was purchased from Sigma (https://www.sigmaaldrich.com) and MC38 was a gift from Dr. Marcus Bosenberg at Yale School of Medicine, CT USA. All cell lines were female sex. AT3 cells were cultured in DMEM–High Glucose medium containing L-glutamine and sodium pyruvate (Sigma Cat. No. D6429) + 1X non-essential amino acids +15 mM HEPES (7.5 mL of 1 M HEPES for 500 mL media) + 1X β-mercaptoethanol (Cat. No. ES-007-E) + 10% FBS and 1% P/S. CT26 cells were cultured in RPMI1640 (+2 mM L-Glutamine) + 10% FBS and 1% P/S. EMT6 cells were cultured in Waymouth (+2mM L-glutamine, Gibco #11220035) + 15% FBS and 1% P/S. MC38 cells were cultured in DMEM/F12 (Gibco #11330032) + 1% non-essential amino acids (Gibco #11140050) + 10% FBS +1% P/S. Cell lines were authenticated by genome sequencing and comparison to previously reported genotypes. Cell lines were tested and verified to be free of mycoplasma contamination.

### METHOD DETAILS

#### Tumor growth and ICI efficacy studies

To measure tumor growth and assess ICI efficacy, tumor cell lines were injected into female mice aged 8–12 weeks. For MC38 and CT26, 0.5 million cells were injected subcutaneously seven days before ICI dosing. For AT3 and EMT6, cells were injected orthotopically into mammary fat pads at a dosage of 0.3 million (EMT6) or 1 million (AT3) cells either seven (EMT6) or ten (AT3) days before ICI dosing. During the dosing period, either isotype control (Bioxcell, 2A3) or aPD1 antibody (Bioxcell, 29F.1A12) was injected intraperitoneally at 200ug per mouse every three days culminating in a total of four doses. Throughout the study, body weights, tumor measurements, and clinical observations were performed three times per week. Mice were removed from a study and euthanized if animal care staff noted tumor ulceration, body weight loss >20%, or any other veterinary care issue. For single dose studies, measurements of body weight and tumor size were performed daily after tumor cell injection. When tumors reached a size of 75-110mm^3^, a single dose of isotype control or aPD1 antibody was administered and the mice were euthanized 48 h later.

#### Blocking antibody experiments

For blocking antibody experiments, mice were treated with aGM-CSF (clone MP1-22E9, Bioxcell, 100ug i.p.), aIL2RB (clone TM-Beta 1, BioXcell, 200ug i.p.), or corresponding isotype controls (same isotype and dose amount as corresponding blocking antibody, Bioxcell) starting immediately after tumor engraftment and given every third day until tumors reached 75-110mm^3^. Treatment groups consisted of mice treated with isotype controls, aPD1+blocking antibody isotype controls, aGM-CSF+aPD1, aIL2RB + aPD1, and aGM-CSF+aIL2RB + aPD1.

#### Whole genome sequencing

High-molecular weight DNA and RNA was extracted from frozen cell pellets using AllPrep DNA/RNA/miRNA Universal kit (Qiagen) according to the manufacturer’s protocol. Genomic DNA libraries of 450bp insert size were prepared using the TruSeq DNA PCR-free Library Preparation Kit (Illumina) according to manufacturer guidelines and sequenced to at least 30x coverage by the New York Genome Center (NYGC).

#### Digestion of tumor samples

Mice were euthanized via cervical dislocation, tumors were excised and separated from surrounding skin using a razor blade. Tumors were split into 2mm wide strips (if necessary) and were submerged in 10% DMSO (Millipore Sigma) in DMEM (ThermoFisher Scientific) and gradually frozen in isopropanol at −80°C overnight before storage in liquid nitrogen.

For analysis, samples were thawed to 37°C in a water bath and rinsed in “FACS buffer” (1x Ca^2+^ and Mg^2+^ deficient HBSS (ThermoFisher Scientific) supplemented with 1% BSA (Research Products International)). The samples were thoroughly minced into <0.5mm pieces using a sterile razor blade and digested in 6mL “digest solution” (1x HBSS with Ca^2+^ and Mg^2+^ HBSS (ThermoFisher Scientific) supplemented with 1% BSA, Collagenase VIII (Millipore Sigma) at 2 mg/mL, and DNase I (Millipore Sigma) at 50ug/mL). Samples were incubated at 37°C and shaken at 250RPM for 30 min. Samples were Q.S. to 20mL with FACS buffer, filtered through a 100μm strainer, and centrifuged at 500 g at 4°C for 10 min. After decanting digest solution, samples were resuspended in 5mL Hybri-Max Red Blood Cell Lysis (Millipore Sigma) for 7 min before Q.S. to 30mL with FACS buffer and centrifuged again before sample processing.

#### Cell staining for FACS

Cells were stained for 30 min on ice in the dark with an MHC Class I Tetramer (PE) (KSPWFTTL, NIH Tetramer core) recognizing an MC38 antigen-specific T cell receptor (TCR), PE-CF594-CD3e (clone 145-2C11; BD Biosciences, San Jose, CA), PerCP-Cy5.5-CTLA4 (UC10-4B9; Biolegend, Inc., San Diego, CA), PE-Cy7-F4/80 (BM8.1; Biolegend, Inc., San Diego, CA), APC-MHCII (M5/114.15.2; Biolegend, Inc., San Diego, CA), AF700-CD206 (C068C2; Biolegend, Inc., San Diego, CA), AFC-AF750-Ly6C (HK1.4; Biolegend, Inc., San Diego, CA), BV421-PD1 (RMP1-30; Biolegend, Inc., San Diego, CA), BV510-CD4 (GK1.5; Biolegend, Inc., San Diego, CA), BV570-CD45 (30-F11; Biolegend, Inc., San Diego, CA), BV605-PDL1 (MIH6; Biolegend, Inc., San Diego, CA), BV650-CD11b (M1/70; BD Biosciences, San Jose, CA), BV711-CD8a (53–6.72; BD Biosciences, San Jose, CA), BV786-Ly6G (1A8; Biolegend, Inc., San Diego, CA), FC Block (anti-CD16/32 (clone 2.4G2; Lienco Technologies, Inc., Fenton, MO)) and Brilliant Stain Buffer Plus (BD Biosciences) in FACS buffer. Samples were washed twice by Q.S. to 1mL and resuspension, centrifuging at 500g for 10 min at 4°C, and decanting the liquid. Samples were then resuspended in 250uL 1:1000 YO-PRO (ThermoFisher) in FACS buffer and filtered through a 35μm strainer into FACS tubes on ice in the dark (centrifuge at 500g for 20 s, if necessary, to pass cells through strainer). DAPI (ThermoFisher Scientific) was added and each sample was vortexed for 3 s before immediate detection on a FACSymphony A5 SE (BD Biosciences). Samples were analyzed with FlowJo software (BD Biosciences).

#### Bulk RNA-Seq data generation

For bulk RNA-Seq samples, we harvested tumors and froze in DMEM/10% FCS with 10% DMSO. We isolated RNA using the RNeasy (QIAGEN) RNA extraction kit. Poly(A) RNA-seq libraries were constructed using the KAPA mRNA HyperPrep Kit (KAPA Biosystems). Libraries were sequenced 100 bp paired-end on the NovaSeq 6000 (Illumina) using the S4 Reagent Kit (Illumina). We constructed transcriptomes for each CC founder strain using GENCODE vM23 annotations by using g2gtools v0.2.9 (https://github.com/churchill-lab/g2gtools) to alter sites that differed from the mouse reference genome according to Mouse Genomes Project SNP and indel release version 5.^86^ We mapped reads to these transcriptomes using bowtie v1.2.3,^78^ followed by converting to EMASE format and using gbrs v0.1.6,^79^ which can account for reads mapping to multiple or divergent haplotypes, to quantify multiway expression across the eight founder haplotypes. We used only total expected read counts across each gene for our analyses.

#### Single cell RNA-Seq data generation

Dissociated tumor cells from responder and non-responder mice strains were incubated with FcR blocking solution, then stained with cell hashing antibodies (TotalSeq-C anti-human Hashtag (HTO) C0301-C0304, BioLegend) and with BV570-CD45 (clone 30-F11), Brilliant Stain Buffer Plus in FACS buffer in the dark on ice for 30 min. After a FACS buffer rinse, samples were then resuspended in 1:1000 YO-PRO in FACS Buffer and filtered through a 35μm strainer into FACS tubes on ice in the dark (centrifuge at 500g for 20 s, when necessary, to pass cells through strainer). DAPI was added and each sample was vortexed for 3 s before immediate sorting (FACSAria, BD Biosciences). Combined samples stained with different hashtags were sorted into the same collection tube for single cell RNA-Seq. From the total viable cells in each sample (DAPI^−^YOPRO1^−^), 90% CD45^+^ and 10% CD45^−^ cells were sorted into 5μL FBS per collection tube, keeping the concentration from each sample equal in each tube if possible. Cell viability was assessed with Trypan Blue on an automated cell counter (Countess II, ThermoFisher), and up to 20,000 cells (~5,000 cells from each hashtagged sample) were loaded onto one lane of a 10x Genomics chip, then cells and gel beads were portioned in a Chromium X instrument (10x Genomics). Library preparation was performed using NextGEM single cell 5′ version 2 chemistry and according to the manufacturer’s protocol (10x Genomics, CG000330). cDNA and libraries fragments were profiled by automated electrophoresis (Tapestation 4200, Agilent) and High Sensitivity D5000 reagents and fluorometry using High Sensitivity 1x dsDNA reagents (Qubit, Invitrogen) and the presence of Illumina adapters was verified via qPCR (QuantStudio 7 Flex, Applied Biosystems). Libraries were then pooled using a ratio of 90% gene expression library and 10% HTO library before sequencing; each gene expression-HTO library pair was sequenced at 15% of an Illumina NovaSeq 6000 S4 v1.5 200 cycle flow cell lane, with a 28-10-10-90 asymmetric read configuration, targeting 10,000 barcoded cells with an average sequencing depth of 75,000 reads per cell.

#### Fresh frozen Visium spatial transcriptomics data generation

After resection, each tissue was blotted dry with an RNase-free laboratory towel then immediately submerged in fresh isopentane chilled over liquid nitrogen for approximately 1 min and transferred to a cryovial pre-cooled on dry ice for long term storage at −80C. Tissue was subsequently submerged in Optimal Cutting Temperature compound (OCT) in a prechilled cryomold using prechilled forceps, and the cryomold was immediately placed on powdered dry ice and allowed to completely freeze. OCT blocks were stored at −80C until quality control and processing. For spatial transcriptomics, five 10μm sections from each block were used for total RNA extraction (RNeasy Mini Kit, Qiagen) and determination of RNA integrity (RINe score) using an automated electrophoresis (Tapestation 4200, Agilent) and High Sensitivity RNA ScreenTape. Blocks with RINe greater than 7 were optimized for ideal permeabilization time following the vendor protocol (10x Genomics, CG000238). Sections from four tissue blocks were placed on Visium Gene Expression slide for H&E staining and brightfield imaging via NanoZoomer SQ (Hamamatsu), block-specific tissue permeabilization, mRNA capture, and subsequent library generation per the manufacturer’s protocol (10x Genomics, CG000239). Libraries were quantified by automated electrophoresis (Tapestation 4200, Agilent) and High Sensitivity D5000 reagents and fluorometry (Qubit, Invitrogen) and the presence of Illumina adapters was verified via qPCR (QuantStudio 7 Flex, Applied Biosystems). Libraries passing quality control were pooled for sequencing on an Illumina NovaSeq 6000 200 cycle S4 flow cell using a 28-10-10-90 read configuration, targeting 100,000 read pairs per spot covered by tissue. Illumina base call files for all libraries were converted to FASTQs using bcl2fastq v2.20.0.422 (Illumina). Whole Visium slide images were uploaded to a local OMERO server. For each capture area of the Visium slide, a rectangular region of interest (ROI) containing just the capture area was drawn on the whole slide image via OMERO.web and OMETIFF images of each ROI were programmatically generated using the OMERO Python API.

### QUANTIFICATION AND STATISTICAL ANALYSIS

#### Genome, mutational signature, and tumor mutational burden (TMB) analysis

The sequencing data for all four cell lines was processed and aligned to the mouse genome (mm10) through NYGC’s variant somatic pipeline v6 (https://bioinformatics.nygenome.org/wp-content/uploads/2019/06/SomaticPipeline_v6.0_Human_WGS.pdf). In brief, reads were aligned to the mouse genome (mm10) using the Burrows-Wheeler Aligner (BWA).^70^ Structural variant (SV) calls were generated using three different tools (Crest,^71^ Delly,^72^ and BreakDancer^73^), and high confidence events were selected when called by at least two tools and by requiring split-read support. Single Nucleotide Variants were called by muTect,^74^ Strelka^75^ and LoFreq.^76^ We considered high confidence mutations as those identified by all three tools. The reference C57BL/6 genome was used as a matched normal for MC38 and AT3 while the BALB/cJ genome was used for CT26 and EMT6. Somatic mutations were considered those unique to each cell line and absent from an in-house generated panel of normal samples (PON) comprising 11 normal mouse genomes. In addition, high-confidence SVs were identified by filtering out SVs detected in an in-house cohort of 63 mouse cancer genomes and those only detected by one SV caller or not supported by split reads, to reduce possible germline events and sequencing artifacts. Tandem duplicator Phenotype (TDP) was assessed as described elsewhere.^87^ We ran deconstructSigs (v1.8.0)^77^ on the high confidence somatic SNV call set within autosomes to estimate the contribution of known COSMIC mutational signatures (v1-2013)^88^ in the tumor sample. TMB was defined as the number of somatic, coding, SNVs and short indels per megabase of genome examined.^89^ Mutations that are present in dbSNP (dbSNP 142)^90^ were excluded from the analysis.

#### Quantification of tumor growth rates

In order to calculate a quantitative measure of the mean and variance in response to aPD1 immunotherapy, we devised a per-mouse metric to measure tumor growth and regression after treatment. We developed this metric within the framework of the treatment/control tumor volume ratio, which is widely used in tumor xenograft studies. Hather et al.^39^ showed that a summary metric they called the rate-based T/C is a straightforward and powerful method for estimating treatment efficacy in such a study. This method makes the assumption that tumor volume follows an exponential growth trajectory and fits a linear regression model to derive a growth rate for each tumor from the logarithm (base 10) of tumor volumes. The rate-based T/C is computed as Rate-based $T\;∕\;C=10^{(\mu_{T}-\mu_{C})\times 21\;\mathrm{days}}$, where $\mu_{T}$ is the mean of the tumor growth rates for the treatment group, and $\mu_{C}$ is the mean of the tumor growth rates for the control group.^39^ In effect, this method compares the slopes of log-transformed tumor volume measurements through the study period and computes the ratio of these numbers as a measure of growth inhibition in the treated group compared to the control group. We adapted the rate-based T/C to apply to our syngeneic host model, computing these quantities separately within each genetically distinct line (e.g., CCF1, B6, BALB). Rather than computing group means for the treated and control groups, we computed per-mouse T/C ratios by extracting tumor growth curve slopes for each individual mouse and normalizing by dividing by the mean slope in the same line’s control group. Therefore, our metric is computed using the formula: per-mouse rate-based $T\;∕\;C=10^{(b_{i}-\mu_{C})\times 21}$, where $b_{i}$ denotes the tumor growth rate of mouse i. When computing slopes from log-transformed tumor volumes, we considered data starting from the first day of ICI dosing (e.g., day 7 for MC38), and omitted measurements of tumors below detectable size (because the logarithm of zero is undefined). For tumors that initially grew but quickly regressed to zero after a single non-zero measurement, we inserted a small value for tumor growth slope in order to capture this responsiveness to ICI. Specifically, we used the 10^th^ quantile of all (log-transformed) tumor growth slopes, computed separately in each treatment group (isotype or aPD1). For QTL mapping, we mapped the mean of the logarithm of per-mouse rate-based T/C computed for each CCF1 line.

Finally, we found that a small number of lines appeared to have an enrichment in the aPD1 treated group of mice carrying tumors that never grew (complete responder*; CR* tumors). Because this enrichment was specific to the aPD1 treatment group, it likely represented tumors that were super responders but could not be registered using the RTC methology. We used the binomial exact test to quantify how unusual the enrichment of CR* tumors was in each CCF1 line. Two CCF1 lines (CC75 and CC68) showed significant enrichment of CR* tumors in aPD1 compared to isotype control group. In order to include this component of aPD1 response in our mapping, we used the large sample binomial approximation to obtain an approximate *Z* score for this test and computed a weighted average with the RTC metric (first converting to a *Z* score) using weights 20% and 80% because approximately 20% of lines had >1 CR* in the aPD1 treatment group. The results of mapping this combined trait were substantially similar to mapping of RTC alone, and the peak on Chr15 (main text) had a marginally more significant LOD score (data not shown).

For CCF1N1 mapping studies, we could not compute the per-mouse rate-based T/C described above because each mouse was genetically unique and there was no isotype control group for normalization. Instead, we mapped the un-normalized quantity (slope of the log-transformed tumor growth curve). We also mapped two additional traits consisting of discrete classifications of response. First, we mapped the binary indicator of whether (or not) each mouse’s tumor was a complete responder (CR) to treatment. Second, we mapped a more granular classification of tumor response, namely whether each tumor was a non-responder, partial responder, CR, or CR*. In the main text we present results of mapping the binary CR indicator because this method was simple and utilized all mice. Nevertheless, all QTL we report in the main text were prominent and statistically significant in at least two of the three traits mapped.

#### Heritability estimation

To estimate heritability of immunotherapy response, we used the rate-based T/C values described above. We rankZ-normalized raw rate-based T/C values which, when these values were regressed against strain, produced residuals that were approximately normally distributed. We then estimated broad-sense heritability using an ANOVA framework where the heritability was estimated using the formula $MSE_{strain}∕(MSE_{strain}+(n-1)\times MSE_{residuals})$ where MSE signifies mean squared error from the ANOVA analysis, and n indicates the mean sample size across all strains. To quantify the significance of heritability estimates, we shuffled strain labels and recomputed heritability (*n* = 10000 times) to build an empirical null distribution, which we used to calculate *p*-values for the test of whether heritability was significantly greater than chance. To obtain confidence intervals for heritability estimates, we recomputed heritability on *n* = 10000 bootstrap samples of the data.

#### Quantitative trait locus mapping

To map quantitative trait loci (QTLs) that harbor genetic elements mediating variation in the response to aPD1 immunotherapy, we considered our CCF1 lines as a genetic mapping population. We genotyped one female and one male mouse of each CC strain using the GigaMUGA array.^91^ We compared our data to previously reported strain genotypes derived from mice sourced from a different institution^92^ and found high agreement, with the closest match to each strain having the same strain designation. We used in-house female genotypes for QTL mapping since we performed aPD1 testing in female mice. To conduct QTL mapping we utilized the R/qtl2 package^93^ using the “genail” cross type. To initialize mapping we followed https://github.com/rqtl/qtl2data/blob/master/CC/R/convert_cc_data.R and obtained the necessary funnel codes describing the breeding scheme for this genetic resource population from Shorter et al.^94^ We mapped the specific phenotypes described above (“Quantification of tumor growth rates”) and used a permutation test to establish statistical significance as implemented in the qtl2 function scan1perm, with the following parameters: 1000 permutations and significance threshold 0.05.

To test for epistasis among QTL, we used logistic regression modeling of data from our CCF1N1 crosses. Specifically, we modeled a binary indicator of whether (or not) each mouse’s tumor was a complete responder to treatment. In the logistic regression framework, this outcome was modeled as a function of QTL ancestry (there are two possible genotypes at each QTL in the CCF1N1 framework), where we modeled either additive or interactive effects of each QTL. We obtained *p*-values for epistatic interactions by performing likelihood ratio tests comparing models with interactive effects to models with only additive effects. To test for differences between groups of mice carrying ancestry at particular loci (e.g., Figure 2D), we performed Wilcoxon tests comparing the fraction of mice in each group that were complete responders.

#### Bulk RNA-Seq analysis

For differential expression testing, we used the DESeq2 package v1.44.0^80^ with default options and following author recommendations. For KEGG pathway enrichment, we used the enrichKEGG function in the clusterProfiler package.^95^ For the heatmap produced in Figure 4E, we performed differential expression testing on all pairwise combinations of groups in our blocking antibody experiment, identified enriched Gene Ontology biological process pathways using log2FoldChange values and the fgsea package v1.30.0 (https://doi.org/10.1101/060012), pruned to semantically distinct terms (Wang measure, threshold 0.6) using GOSemSim v2.30.0,^81^ and filtered to immune-related pathways by considering only terms descended from the following list of immune-related GO terms: “immune response”, “immune system process”, “response to cytokine”, “regulation of immune system process”. For the principal components analysis (PCA) depicted in Figure 4D, we started with bulk RNA-Seq samples from (1) genetic responders and non-responders (2) our blocking antibody experiment. We normalized gene expression using edgeR v4.2.0,^96^ and leveraged the ComBat method^82^ to minimize batch effects between these two sets of samples. Finally we used DESeq2 as above to identify differentially expressed genes between responders and non-responders, and subset the matrices to this st of 2,324 genes that were differentially expressed at FDR = 5% and quantified in both RNA-Seq experiments. We used these subsetted, batch-adjusted matrices for PCA. For deconvolution of bulk RNA-Seq samples in the blocking antibody experiment, we used mMCP-Counter^83^ with default parameters. To compare the *Cxcl9/Spp1* ratio between groups, we computed this quantity for each RNA-Seq sample and compared using the Wilcoxon test.

#### Single cell RNA-Seq analysis

Illumina base call files for all libraries were converted to FASTQs using bcl2fastq v2.20.0.422 (Illumina) and FASTQ files associated with the gene expression libraries were aligned to the GRCm38 reference assembly with vM23 annotations from GENCODE (10x Genomics mm10 ref. 2020-A) using the version 6.1.1 Cell Ranger count pipeline (10x Genomics). We carried out analyses of processed scRNA-Seq data in R version 4.3.3,^97^ using Seqgeq software (BD Biosciences), and the R package Seurat version 5.0.1.^84^ We quantified expression across 32,285 genes, of which 26,938 were expressed in at least one cell. After filtering out cells with >15% mitochondrial gene expression or >45% ribosomal gene expression, we obtained data from 93,126 cells passing quality control steps. We normalized using log normalization and selected 2,000 variable features. We scored cells according to cell cycle following documentation available with Seurat. We scaled/centered feature expression after regressing out the total read count, percent mitochondrial gene expression, percent ribosomal gene expression, and cell cycle phase of each cell. We reduced dimensionality using principal components analysis (PCA) and used harmony v0.1^85^ with 37 PCs using option theta = 1 to correct for batch processing date of our scRNA-Seq libraries. for cell clustering. We used the shared nearest neighbor-based modularity optimization algorithm implemented in Seurat to cluster cells, using resolution 0.1, and projected into UMAP space using 37 PCs. For subclustering of monocyte/macrophages, we reanalyzed these cells and clustered with 26 PCs and using clustering resolution 0.6. For T/NK cells we performed a similar analysis but using 19 PCs and clustering resolution 0.3. To compare cell cluster/subcluster abundance between groups, we used the Wilcoxon test performed on the fraction of cells assigned to the cluster of interest for each mouse sample.

#### Spatial transcriptomics analysis

FASTQ files and associated OMETIFF corresponding to each capture area were aligned to the GRCm38 reference assembly with vM23 annotations from GENCODE (10x Genomics mm10 ref. 2020-A) using the version 1.3.1 Space Ranger count pipeline (10x Genomics). We carried out analyses of processed spatial transcriptomics data in R version 4.3.3,^97^ using Seqgeq software (BD Biosciences), and the R package Seurat version 5.0.1.^84^ We normalized spot gene expression using the SCTransform algorithm implemented in Seurat. To test for colocalization of cell types: cytotoxic T lymphocytes (CTLs) with dendritic cells (DCs) or *Cxcl9*^*+*^ macrophages) we computed binary indicators of nonzero expression of the marker genes mentioned (main text) for each spot and deemed two cell types as colocalized within a spot if markers of both cell types were expressed in that spot. To test for differences between colocalization (“CTL-DC” or “CTL-mac”), we developed a resampling procedure that leveraged the thousands of spatial transcriptomic spots profiled. Each spatial transcriptomic spot is associated with a sample and either showed or did not show evidence of colocalization for the two relevant cell types (CTL-DC or CTL-mac). To test for differences in colocalization between responders and non-responders, we shuffled the sample labels for all spots and then recomputed the difference in colocalization between the (shuffled) responder and non-responder samples. For this test, a *p*-value is computed as the fraction of shuffled datasets with a responder/non-responder difference that is as or more extreme than the observed value.

## Supplementary Material

Doc S1

Table S1

Supplemental information can be found online at https://doi.org/10.1016/j.celrep.2025.115698.

## Figures and Tables

**Table:** KEY RESOURCES TABLE

REAGENT or RESOURCE	SOURCE	IDENTIFIER
Antibodies		
Hamster anti-mouse CD3e PE-CF594 (clone 145-2C11)	BD Biosciences	CAT#562286; RRID:AB_11153307
anti-mouse CD152 (CTLA-4) PerCp-Cy5.5 (clone UC10-4B9)	Biolegend	CAT#106316; RRID:AB_2564474
anti-mouse Ly71 (F4/80) PE-Cy7 (clone BM8.1)	Biolegend	CAT#123114; RRID:AB_893478
anti-mouse I-A/I-E (MHC Class II) APC (clone M5/114.15.2)	Biolegend	CAT#107614; RRID:AB_313329
anti-mouse CD206 (MMR) AF700 (clone C068C2)	Biolegend	CAT#141734; RRID:AB_2629637
anti-mouse Ly-6C AFC-AF750 (clone HK1.4)	Biolegend	CAT#128046; RRID:AB_2616731
anti-mouse CD279 (PD-1) BV421 (clone RMP1-30)	Biolegend	CAT#109121; RRID:AB_2687080
anti-mouse CD4 BV510 (clone GK1.5)	Biolegend	CAT#100449; RRID:AB_2564587
anti-mouse CD45 BV570 (clone 30-F11)	Biolegend	CAT#103136; RRID:AB_2562612
anti-mouse CD274 (BH-H1, PD-L1) BV605 (clone MIH6)	Biolegend	CAT#153606; RRID:AB_2814056
Rat anti-CD11b BV650 (clone M1/70)	BD Biosciences	CAT#563015; RRID:AB_2737951
Anti-Human/Mouse CD11b APC-Cy7 (clone M1/70)	Tonbo Biosciences	CAT#25-0112; RRID:AB_2621625
anti-mouse CD8a BV711 (clone 53-6.7)	Biolegend	CAT#100759; RRID:AB_2563510
anti-mouse Ly-6G BV786 (clone 1A8)	Biolegend	CAT#127645; RRID:AB_2566317
anti-mouse CD16/CD32 (clone 2.4G2)	Leinco Technologies, Inc.	CAT#C381-1.0 mg; RRID:AB_2737484
H-2K(b)/MuLV.p15E.KSPWFTTL PE labeled tetramer	NIH Tetramer Facility	N/A
TotalSeq^™^-C0301 anti-mouse Hashtag 1 Antibody (clone M1/42; 30-F11)	Biolegend	CAT#155861; RRID:AB_2800693
TotalSeq^™^-C0302 anti-mouse Hashtag 2 Antibody (clone M1/42; 30-F11)	Biolegend	CAT#155863; RRID:AB_2800694
TotalSeq^™^-C0303 anti-mouse Hashtag 3 Antibody (clone M1/42; 30-F11)	Biolegend	CAT#155865; RRID:AB_2800695
TotalSeq^™^-C0304 anti-mouse Hashtag 4 Antibody (clone M1/42; 30-F11)	Biolegend	CAT#155867; RRID:AB_2800696
InVivoPlus anti-mouse PD-1(CD279) (Clone 29F.1A12)	Bio X Cell	CAT#BP0273; RRID:AB_2687796
InVivoPlus rat IgG2a isotype control, anti-trinitrophenol (Clone 2A3)	Bio X Cell	CAT#BP0089; RRID:1107769
*InVivo*MAb anti-mouse GM-CSF (clone MP1-22E9)	Bio X Cell	CAT#BE0259; RRID:AB_2687738
*InVivo*MAb anti-mouse CD122 (IL-2Rβ) (clone TM-Beta 1)	Bio X Cell	CAT#BE0298; RRID:AB_2687820
*InVivo*MAb rat IgG2b isotype control, anti-keyhole limpet hemocyanin (clone LTF-2)	Bio X Cell	CAT#BE0090; RRID:AB_1107780
Chemicals, peptides, and recombinant proteins		
YO-PRO-1 Iodide (491/509) dye	ThermoFisher Incorporated (Invitrogen)	CAT#Y3603
Deposited data		
MC38 responder and non-responder CCF1 RNA-Seq	This paper	GEO: GSE273487
MC38 blocking antibody RNA-Seq	This paper	GEO: GSE273488
MC38 spatial transcriptomics	This paper	GEO: GSE273489
MC38 single cell RNA-Seq	This paper	GEO: GSE274421
Tumor cell line genome sequences	This paper	BioProject: PRJNA1145300
Mouse reference GRCm38/mm10 genome sequence	GENCODE	https://www.gencodegenes.org/mouse/release_M23.html
GENCODE vM23 mouse genome annotations	GENCODE	https://www.gencodegenes.org/mouse/release_M23.html
Experimental models: Cell lines		
AT3	Sigma Aldrich	SCC178
CT26	ATCC	CRL-2638
EMT6	ATCC	CRL-2755
MC38	Dr. Marcus Bosenburg (Yale University)	N/A
Experimental models: Organisms/strains		
C57BL/6J	The Jackson Laboratory	https://www.jax.org/strain/000664; RRID:IMSR_JAX:000664
BALB/cJ	The Jackson Laboratory	https://www.jax.org/strain/000651; RRID:IMSR_JAX:000651
CC001	The Jackson Laboratory	https://www.jax.org/strain/021238; RRID:IMSR_JAX:021238
CC002	The Jackson Laboratory	https://www.jax.org/strain/021236; RRID:IMSR_JAX:021236
CC003	The Jackson Laboratory	https://www.jax.org/strain/021237; RRID:IMSR_JAX:021237
CC004	The Jackson Laboratory	https://www.jax.org/strain/020944; RRID:IMSR_JAX:020944
CC005	The Jackson Laboratory	https://www.jax.org/strain/020945; RRID:IMSR_JAX:020945
CC007	The Jackson Laboratory	https://www.jax.org/strain/029625; RRID:IMSR_JAX:029625
CC008	The Jackson Laboratory	https://www.jax.org/strain/026971; RRID:IMSR_JAX:026971
CC009	The Jackson Laboratory	https://www.jax.org/strain/018856; RRID:IMSR_JAX:018856
CC010	The Jackson Laboratory	https://www.jax.org/strain/021889; RRID:IMSR_JAX:021889
CC011	The Jackson Laboratory	https://www.jax.org/strain/018854; RRID:IMSR_JAX:018854
CC012	The Jackson Laboratory	https://www.jax.org/strain/028409; RRID:IMSR_JAX:028409
CC015	The Jackson Laboratory	https://www.jax.org/strain/018859; RRID:IMSR_JAX:018859
CC016	The Jackson Laboratory	https://www.jax.org/strain/024684; RRID:IMSR_JAX:024684
CC017	The Jackson Laboratory	https://www.jax.org/strain/022870; RRID:IMSR_JAX:022870
CC023	The Jackson Laboratory	https://www.jax.org/strain/025131; RRID:IMSR_JAX:025131
CC024	The Jackson Laboratory	https://www.jax.org/strain/021891; RRID:IMSR_JAX:021891
CC025	The Jackson Laboratory	https://www.jax.org/strain/018857; RRID:IMSR_JAX:018857
CC027	The Jackson Laboratory	https://www.jax.org/strain/025130; RRID:IMSR_JAX:025130
CC032	The Jackson Laboratory	https://www.jax.org/strain/020946; RRID:IMSR_JAX:020946
CC033	The Jackson Laboratory	https://www.jax.org/strain/025910; RRID:IMSR_JAX:025910
CC036	The Jackson Laboratory	https://www.jax.org/strain/025127; RRID:IMSR_JAX:025127
CC044	The Jackson Laboratory	https://www.jax.org/strain/026426; RRID:IMSR_JAX:026426
CC057	The Jackson Laboratory	https://www.jax.org/strain/024683; RRID:IMSR_JAX:024683
CC058	The Jackson Laboratory	https://www.jax.org/strain/027298; RRID:IMSR_JAX:027298
CC060	The Jackson Laboratory	https://www.jax.org/strain/026427; RRID:IMSR_JAX:026427
CC065	The Jackson Laboratory	https://www.jax.org/strain/023830; RRID:IMSR_JAX:023830
CC068	The Jackson Laboratory	https://www.jax.org/strain/025908; RRID:IMSR_JAX:025908
CC074	The Jackson Laboratory	https://www.jax.org/strain/018855; RRID:IMSR_JAX:018855
CC075	The Jackson Laboratory	https://www.jax.org/strain/027293; RRID:IMSR_JAX:027293
CC078	The Jackson Laboratory	https://www.jax.org/strain/025989; RRID:IMSR_JAX:025989
IL6411(CC079)	The Jackson Laboratory	https://www.jax.org/strain/025990; RRID:IMSR_JAX:025990
IL6573(CC080)	The Jackson Laboratory	https://www.jax.org/strain/025988; RRID:IMSR_JAX:025988
Software and algorithms		
FlowJo	BD Biosciences	RRID:SCR_008520; https://www.flowjo.com/
FACSDiva	BD Biosciences	RRID:SCR_001456
Seqgeq	BD Biosciences	https://www.flowjo.com/solutions/seqgeq
Burrows-Wheeler Aligner (BWA)	Li and Durbin 2009^70^	https://bio-bwa.sourceforge.net/
Crest	Wang et al. 2011^71^	http://www.stjuderesearch.org/site/lab/zhang
Delly	Rausch et al. 2012^72^	https://github.com/dellytools/delly
Breakdancer	Chen et al. 2009^73^	https://github.com/genome/breakdancer
muTect	Cibulskis et al. 2013^74^	http://www.broadinstitute.org/cancer/cga/mutect
Strelka	Saunders et al. 2012^75^	https://github.com/genome-vendor/strelka
LoFreq	Wilm et al. 2012^76^	https://csb5.github.io/lofreq/
deconstructSigs v1.8.0	Rosenthal et al. 2016^77^	https://github.com/raerose01/deconstructSigs
g2gtools v0.2.9	Churchill laboratory https://churchilllab.jax.org/	https://github.com/churchilllab/g2gtools
bowtie v1.2.3	Langmead et al. 2009^78^	https://bowtie-bio.sourceforge.net/index.shtml
gbrs v0.1.6	Raghupathy et al. 2018^79^	https://gbrs.readthedocs.io/en/latest/index.html
OMERO web	Open Microscopy Environment	https://omeroweb.jax.org/
DESeq2 package v1.44.0	Love et al. 2014^80^	https://bioconductor.org/packages/release/bioc/html/DESeq2.html
fgsea package v1.30.0	Korotkevich et al. 2021 https://doi.org/10.1101/060012	https://bioconductor.org/packages/release/bioc/html/fgsea.html
GOSemSim v2.30.0	Yu 2020^81^	https://bioconductor.org/packages/release/bioc/html/GOSemSim.html
ComBat	Johnson et al. 2007^82^	https://bioconductor.org/packages/release/bioc/html/sva.html
mMCP-Counter	Petitprez et al. 2020^83^	https://github.com/cit-bioinfo/mMCP-counter
Cell Ranger version 6.1.1	10X Genomics	https://www.10xgenomics.com/support/software/cell-ranger/latest
R version 4.3.3	The Comprehensive R Archive Network	https://cran.r-project.org/
bcl2fastq v2.20.0.422	Illumina, Inc.	https://emea.support.illumina.com/downloads/bcl2fastq-conversion-software-v2-20.html
Seurat version 5.0.1	Hao et al. 2021^84^	https://cran.r-project.org/web/packages/Seurat/index.html
harmony v0.1	Korsunsky et al. 2019^85^	https://cran.r-project.org/web/packages/harmony/index.html
Other		
Code to reproduce tumor growth quantification, mapping results, functional genomics analyses, and survival analyses	This paper	https://doi.org/10.6084/m9.figshare.c.7398247

## Highlights

- Host genetic variation strongly influences response to aPD1
- Genetic and genomic analyses identify host genes predicting aPD1 response
- Blockade of two of these genes abrogates aPD1 response
