# Supplementary material for: Mapping the genetic landscape establishing a tumor immune microenvironment favorable for anti-PD-1 response
Source: Cell Rep. Author manuscript; Available in PMC 2025 Sep 2. (PMC12404271; doi:10.1016/j.celrep.2025.115698)
Supplement: Doc S1 [file NIHMS2104859-supplement-Doc_S1.pdf]

**Cell Reports, Volume *44***

## Supplemental information

# Mapping the genetic landscape establishing a tumor immune microenvironment favorable for anti-PD-1 response

**Daniel A. Skelly, John P. Graham, Mingshan Cheng, Mayuko Furuta, Andrew Walter, Thomas A. Stoklasek, Hongyuan Yang, Timothy M. Stearns, Olivier Poirion, Ji-Gang Zhang, Jessica D.S. Grassmann, Diane Luo, William F. Flynn, Elise T. Courtois, Chih-Hao Chang, David V. Serreze, Francesca Menghi, Laura G. Reinholdt, and Edison T. Liu**

**Supplementary Information**

A

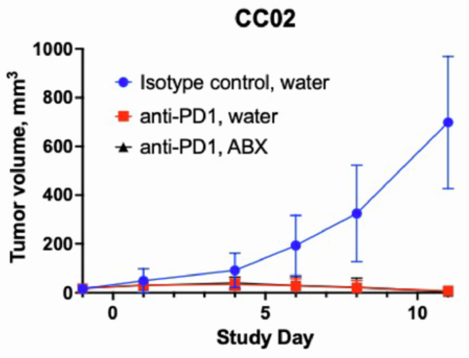


B

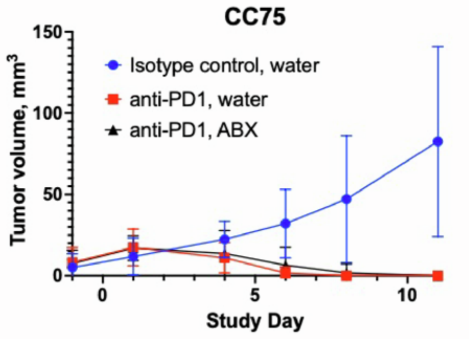


C

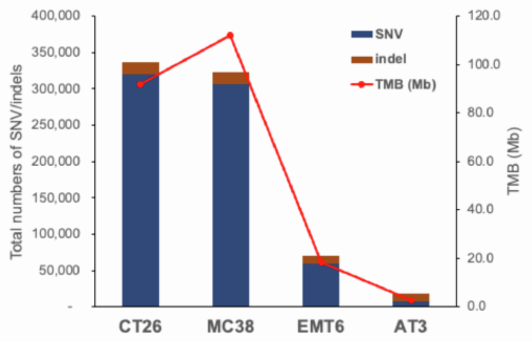


D

| | Deletion | Inversion | Tandem Duplication | Translocation | TDP status |
|---|---|---|---|---|---|
| **CT26** | 165 | 35 | 108 | 31 | TDP_group12mix |
| **MC38** | 93 | 32 | 68 | 15 | TDP_group2 |
| **EMT6** | 82 | 27 | 74 | 12 | TDP_group2 |
| **AT3** | 37 | 12 | 7 | 19 | non TDP |

**Figure S1. Response to immunotherapy in antibiotic-treated mice and genomics of tumor cell lines, Related to Figure 1.** (A-B) Responder strain (CC02 and CC075 F1) mice given broad spectrum antibiotics ("ABX"; vancomycin, streptomycin, ampicillin, and colistin) *ad libitum* in drinking water display no loss in response to PD1 blockade. (C) SNP/indel count and tumor mutation burden (per megabase) in each tumor model. (D) Summary of single base and structural variations, and TDP assessment, quantified in each tumor model.

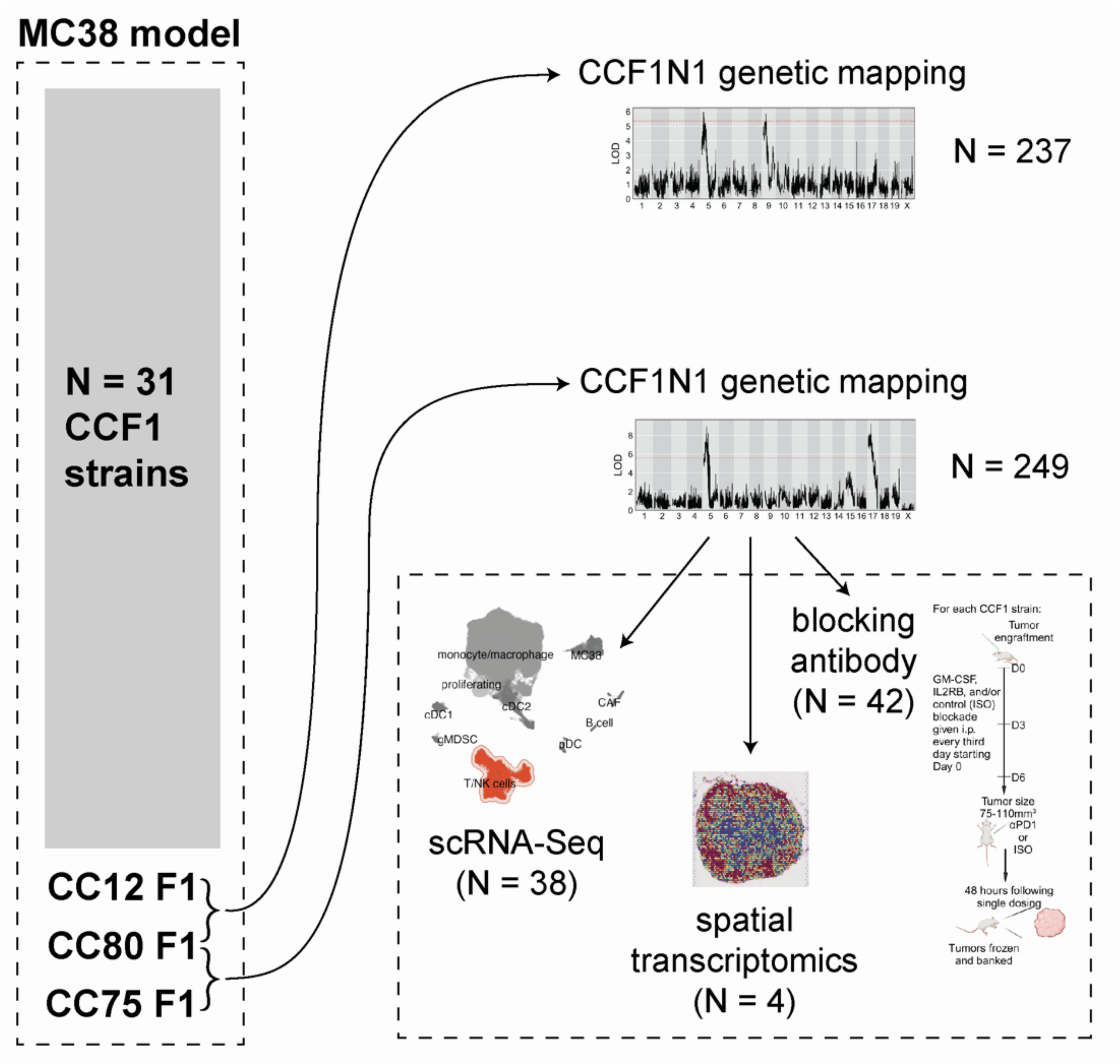


**Figure S2. Schematic of mouse genetic resources used in this study, Related to Figure 1.** At left, box indicates strains used for genetic mapping. Arrows crossing from left to right indicate specific crosses used for CCF1N1 mapping. Downward arrows indicate that mice from the parental lines of this cross were used for further genomic and functional profiling.

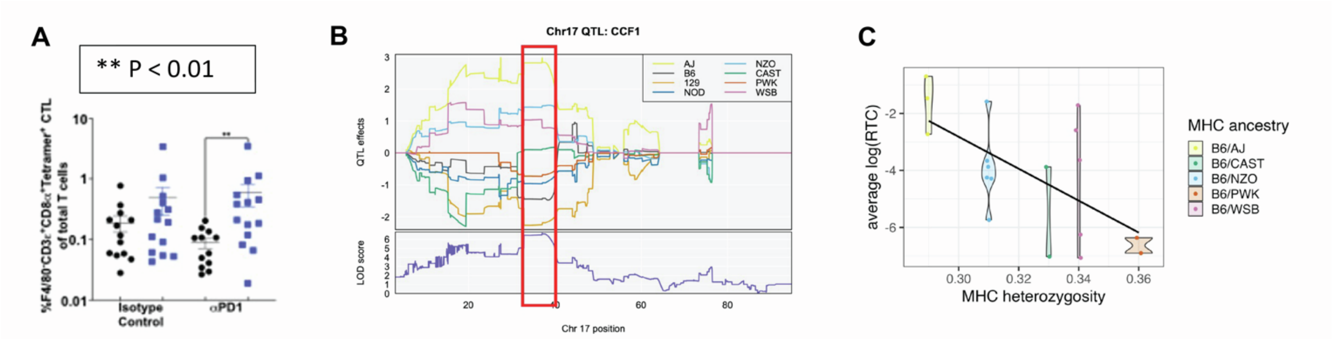


**Figure S3. MC38-specific cytotoxic T lymphocytes (CTL) and MHC genetic variation in immunotherapy response, Related to Figure 4.** (A) FACS data showing MC38-specific CTL as a percentage of total T cells. black circles indicate measurements taken from non-responder strain mice (CC36 F1, CC79 F1, and CC80 F1), blue boxes indicate measurements taken from responder strain mice (CC01 F1, CC02 F1, and CC075 F1). (B) Top panel shows QTL effects plots showing the effect on RTC of carrying a haplotype derived from each of the eight founder parental lines of the CC. Lower number indicates a lower value of RTC, which is associated with better response. Bottom panel shows LOD score across the locus from QTL mapping, as in Figure 2. Red box indicates approximate boundaries of the MHC locus. (C) Plot of ICI response as measured by RTC, stratified by each CCF1 line’s ancestry at the MHC locus, versus MHC heterozygosity. A lower RTC number is associated with better response.

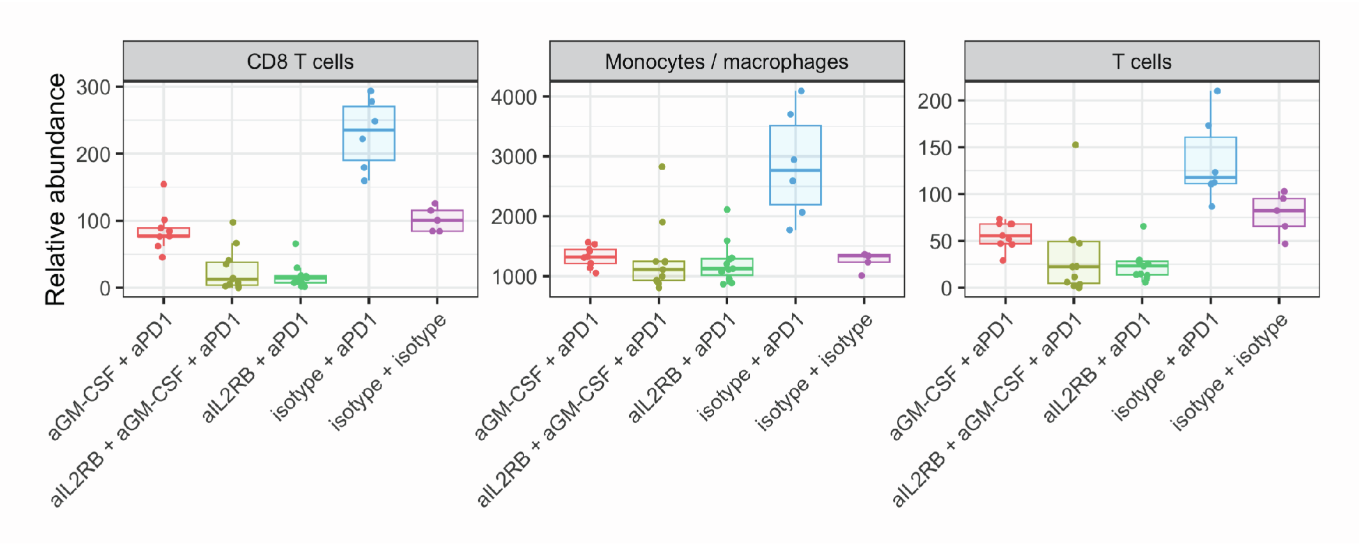


**Figure S4. Bulk RNA-Seq deconvolution of MC38 tumors, Related to Figure 4.**
Deconvolution was performed using mMCP-Counter on samples of MC38 tumors from CC75F1 mice treated with blocking antibodies to PD1, GM-CSF, and/or IL2RB.

| | | Non-responders, αPD1 versus ISO | | | | Responders, aPD1 versus ISO | | | | Responders versus Non-responders (ISO) | | Responders versus Non-responders (αPD1) | |
|---|---|---|---|---|---|---|---|---|---|---|---|---|---|
| Population or cluster | FACS or scRNAseq cluster | ISO | anti-PD1 | Fold change αPD1:ISO | Significant difference? | ISO | anti-PD1 | Fold change αPD1:ISO | Significant difference? | Fold Change R:NR | Significant difference RvsNR? | Fold Change R:NR | Significant difference RvsNR? |
| %CD45$^+$ of Live Cells | FACS | 66.2+/-3.15 | 72.85+/-2.89 | 1.1 | No | 68.16+/-2.82 | 64.69+/-4.41 | 0.95 | No | 1.03 | No | 0.89 | No |
| %macrophages of CD45$^+$ | FACS | 70.38+/-3.03 | 71.03+/-2.11 | 1.01 | No | 68.49+/-2.44 | 69.08+/-2.63 | 1.01 | No | 0.97 | No | 0.97 | No |
| %T cells of CD45$^+$ | FACS | 19.75+/-2.12 | 21.39+/-2.87 | 1.08 | No | 20.57+/-2.28 | 20.11+/-2.47 | 0.98 | No | 1.04 | No | 0.94 | No |
| %CD4$^+$ T cells of total T cells | FACS | 3.39+/-0.79 | 3.22+/-0.62 | 0.95 | No | 2.15+/-0.30 | 2.02+/-0.20 | 0.94 | No | 0.63 | No | 0.63 | No |
| %CTLs of total T cells | FACS | 2.16+/-0.43 | 1.57+/-0.36 | 0.73 | No | 2.82+/-0.55 | 3.37+/-0.74 | 1.2 | No | 1.31 | No | 2.15 | Yes, P*=0.0270 |
| %Tetramer$^+$ CTLs of total T cells | FACS | 0.19+/-0.06 | 0.09+/-0.02 | 0.47 | No | 0.48+/-0.23 | 0.58+/-0.24 | 1.21 | No | 2.53 | No | 6.44 | Yes, P**=0.0037 |
| %Tetramer$^+$PD1$^+$ CTLs of total T cells | FACS | 0.06+/-0.02 | 0.01+/-0.003 | 0.17 | Yes P***=0.0006 | 0.31+/-0.18 | 0.044+/-0.01 | 0.14 | No | 5.17 | No | 3.38 | Yes, P*=0.0108 |
| %CD206$^-$PD-L1$^{high}$MHCII$^{high}$ macrophages of total macrophages | FACS | 12.80+/-1.41 | 17.41+/-3.15 | 1.36 | No | 17.66+/-1.94 | 26.09+/-4.61 | 1.48 | No | 1.38 | Yes, P*=0.0427 | 1.5 | No |
| %CD206$^+$ macrophages of total macrophages | FACS | 31.30+/-3.57 | 23.39+/-3.46 | 0.75 | No | 31.63+/-4.60 | 27.65+/-4.41 | 0.87 | No | 1.01 | No | 1.18 | No |
| %Exhausted CTL of total dataset | 3 | 2.29+/-1.04 | 0.67+/-0.2 | 0.29 | No | 5.041+/-1.17 | 4.81+/-1.2 | 0.95 | No | 2.2 | Yes, P*=0.0435 | 7.18 | Yes, P****<0.0001 |
| %Ifng$^+$ CTL of total dataset | 13 | 0.31+/-0.08 | 0.23+/-0.057 | 0.74 | No | 1.6+/-0.66 | 0.73+/-0.10 | 0.46 | No | 5.16 | Yes, P*=0.0399 | 3.17 | Yes, P**=0.0021 |
| %IFNγ-stimulated macrophages of total dataset | 2, 5, 26 | 3.61+/-0.88 | 3.38+/-0.46 | 0.94 | No | 8.26+/-1.15 | 17.22+/-2.85 | 2.08 | Yes P**=0.0029 | 2.29 | Yes, P**=0.0057 | 5.09 | Yes, P****<0.0001 |
| %Cd209a$^+$ DC of total dataset | 14 | 6.68+/-0.59 | 5.65+/-0.56 | 0.85 | No | 4.44+/-0.62 | 4.21+/-0.58 | 0.95 | No | 0.66 | Yes, P*=0.0172 | 0.75 | No |
| %cDC1 of total dataset | 11 | 1.78+/-0.33 | 1.61+/-0.21 | 0.9 | No | 1.395+/-0.24 | 1.68+/-0.2 | 1.2 | No | 0.78 | No | 1.04 | No |
| %Xcr1$^+$Clec9a$^+$ DC of total dataset | 6 | 1.59+/-0.3 | 1.31+/-0.24 | 0.82 | No | 1.21+/-0.22 | 1.34+/-0.2 | 1.11 | No | 0.76 | No | 1.03 | No |
| %plasmacytoid DC of total dataset | 18 | 0.56+/-0.12 | 0.4+/-0.1 | 0.71 | No | 0.51+/-0.15 | 0.56+/-0.19 | 1.1 | No | 0.91 | No | 1.4 | No |

**Table S2: Overview of flow cytometry results, related to Figure 3.**
